# Application of ^89^Zr-DFO*-immuno-PET to assess improved target engagement of a bispecific anti-amyloid-ß monoclonal antibody

**DOI:** 10.1007/s00259-023-06109-3

**Published:** 2023-01-13

**Authors:** N. Stergiou, T. E. Wuensche, M. Schreurs, I. Mes, M. Verlaan, E. J. M. Kooijman, A. D. Windhorst, L. Helboe, S. Vergo, S. Christensen, A. A. Asuni, A. Jensen, G. A. M. S. Van Dongen, B. Bang-Andersen, D. J. Vugts, W. Beaino

**Affiliations:** 1 Radiology & Nuclear Medicine, Amsterdam UMC location Vrije Universiteit Amsterdam, de Boelelaan 1117, Amsterdam, The Netherlands; 2 H. Lundbeck A/S, Copenhagen, Denmark

**Keywords:** Alzheimer’s disease, Immuno-PET, Aducanumab, Transferrin receptor, Amyloid imaging

## Abstract

**Purpose:**

The recent conditional FDA approval of Aducanumab (Adu) for treating Alzheimer’s disease (AD) and the continued discussions around that decision have increased interest in immunotherapy for AD and other brain diseases. Reliable techniques for brain imaging of antibodies may guide decision-making in the future but needs further development. In this study, we used ^89^Zr-immuno-PET to evaluate the targeting and distribution of a bispecific brain-shuttle IgG based on Adu with transferrin receptor protein-1 (TfR1) shuttling mechanism, mAbAdu-scFab8D3, designated Adu-8D3, as a candidate theranostic for AD. We also validated the ^89^Zr-immuno-PET platform as an enabling technology for developing new antibody-based theranostics for brain disorders.

**Methods:**

Adu, Adu-8D3, and the non-binding control construct B12-8D3 were modified with DFO*-NCS and radiolabeled with ^89^Zr. APP/PS1 mice were injected with ^89^Zr-labeled mAbs and imaged on days 3 and 7 by positron emission tomography (PET). *Ex vivo* biodistribution was performed on day 7, and *ex vivo* autoradiography and immunofluorescence staining were done on brain tissue to validate the PET imaging results and target engagement with amyloid-β plaques. Additionally, [^89^Zr]Zr-DFO*-Adu-8D3 was evaluated in 3, 7, and 10-month-old APP/PS1 mice to test its potential in early stage disease.

**Results:**

A 7-fold higher brain uptake was observed for [^89^Zr]Zr-DFO*-Adu-8D3 compared to [^89^Zr]Zr-DFO*-Adu and a 2.7-fold higher uptake compared to [^89^Zr]Zr-DFO*-B12-8D3 on day 7. Autoradiography and immunofluorescence of [^89^Zr]Zr-DFO*-Adu-8D3 showed co-localization with amyloid plaques, which was not the case with the Adu and B12-8D3 conjugates. [^89^Zr]Zr-DFO*-Adu-8D3 was able to detect low plaque load in 3-month-old APP/PS1 mice.

**Conclusion:**

^89^Zr-DFO*-immuno-PET revealed high and specific uptake of the bispecific Adu-8D3 in the brain and can be used for the early detection of Aβ plaque pathology. Here, we demonstrate that ^89^Zr-DFO*-immuno-PET can be used to visualize and quantify brain uptake of mAbs and contribute to the evaluation of biological therapeutics for brain diseases.

**Supplementary Information:**

The online version contains supplementary material available at 10.1007/s00259-023-06109-3.

## Introduction

Dementia affects around 50 million people worldwide [1], with Alzheimer’s disease (AD) being the most common form, which accounts for up to 80% of the cases [2]. Given the rising prevalence and mortality of AD, coupled with the growing total healthcare costs [3], there is an urgent unmet medical need for effective early diagnosis and treatment of this progressive neurodegenerative disease [4]. The neuropathology of AD is characterized by the extracellular accumulation of amyloid plaques, consisting of the Aβ peptides Aβ40 and Aβ42 generated by the cleavage of amyloid precursor protein (APP), and intra-neuronal deposition of neurofibrillary tangles (NFT) composed of hyperphosphorylated tau protein (p-tau) [5]. The emergence of small molecule amyloid-positron emission tomography (PET) imaging provided a tool for quantification of the amyloid load and thus accurate diagnosis and staging of AD. Additionally, it supported the assessment of the therapeutic effects of anti-amyloid-targeted therapies [6]. However, developing specific and effective AD treatments remains a significant hurdle. The approved AD therapies, like cholinesterase inhibitors (i.e., donepezil) or NMDA receptor antagonists (i.e., memantine), only alleviate the symptoms of the disease and do not target the underlying AD pathology [7]. Therefore, research into future AD treatments mainly targets AD pathology, including the hallmark protein Aβ plaques, neurofibrillary tangles, and, more recently, microglial activation (neuroinflammation) [8]. Clinical development of Aβ targeted therapies based on, in particular, passive immunotherapy using anti-Aβ monoclonal antibodies (mAb) has advanced the most. The exciting recent results of lecanumab (Biogen and Eisai) in a phase 3 trial, showing reduced brain amyloid levels and less decline in a clinical measure of cognition and function in AD patients [9], together with the FDA approval of aducanumab (Aduhelm™) [10, 11], demonstrate the high interest in developing mAb-based therapies for the treatment of AD and other brain diseases. Although a post-approval trial is required to verify that aducanumab provides the assumed clinical benefit, its initial approval shows the potential regulatory pathway to success for any drugs targeting AD’s fundamental pathophysiology [12].

The restricted exchange of macromolecules between the blood and the central nervous system (CNS) due to the blood–brain barrier (BBB) represents a key challenge for brain delivery of peripherally administered therapeutic mAbs [13, 14]. Hence, developing biologicals exploiting the receptor-mediated transcytosis (RMT) mechanism for enhancing brain exposure remains a focus area [15, 16]. Recent preclinical efforts on RMT-based delivery strategies have been made using antibody variable domains that target, bind, and activate brain endothelial cell receptors [17–19]. The most well-studied BBB target for brain delivery is the transferrin receptor 1 (TfR1) [20]. It is highly expressed on cerebral vasculature brain endothelial cells, especially in the microvascular capillary beds, and undergoes constitutive ligand-independent endocytosis [21, 22]. Hence, by exploiting the TfR1 shuttling process, the brain uptake of aducanumab could be increased [23]. Different technologies targeting rodent TfR1 have been developed and tested, including the most investigated antibody clones, OX26 [24] and 8D3 [25].

We engineered a bispecific antibody (mAbAdu-scFab8D3, herein called Adu-8D3) consisting of aducanumab with bivalent binding to human Aβ plaques [26] and with a single chain Fab (scFab) of the 8D3 mAb targeting murine TfR1 [25] attached to the heavy c-terminal [19]. In our study, we used ^89^Zr-immuno-PET to investigate the brain uptake and the Aβ-specific targeting of the bispecific antibody compared to aducanumab. Immuno-PET combines the sensitivity of PET with the specificity of antibodies. ^89^Zr has a long half-life (78.4 h) suitable for imaging larger slow-kinetic molecules like bispecific antibodies. Furthermore, ^89^Zr has favorable characteristics related to availability and physical properties for high-resolution immuno-PET imaging and quantification, as well as broad-scale clinical use [27]. This makes the clinical application of ^89^Zr-immuno-PET more attractive than ^124^I-immuno-PET, which has been explored for amyloid-PET imaging in the pioneering work of the group Syvänen [28–30]. Crucial for the use of ^89^Zr-immuno-PET for studying brain targeting of mAbs has been the recent introduction of the chelator DFO*-NCS as the clinical successor of DFO-NCS for the stable coupling of ^89^Zr to mAbs [31, 32]. Specific targeting of Aβ plaques using ^89^Zr-labeled Adu-8D3 bispecific antibody could only be observed when using DFO*-NCS as a chelator[33].

Here, we evaluated [^89^Zr]Zr-DFO*-NCS labeled Adu-8D3 for Aβ imaging and targeting in a preclinical AD mouse model (APP/PS1 transgenic) with the aims a) to evaluate the bispecific Adu-8D3 as a candidate theranostic agent for diagnosis and therapy of AD, and b) to validate the ^89^Zr-immuno-PET platform as an enabling technology for the development of new antibody-based theranostics for brain exposure and target engagement.

## Material and methods

Methods, including general materials, antibody design, cell culture, antibody purification, DFO*-NCS modification, ^89^Zr labeling, quality controls, [^11^C]PIB synthesis, ELISA, and flow cytometry, are available in the supplementary information.

### Antibody constructs

All antibodies are produced by standard methods for antibody production (Table 1). The human monospecific antibody mAbAdu (Adu) targets Aβ plaques but not monomers [10]. The bispecific antibody Adu-8D3 was designed with the rat scFab 8D3 to target the murine TfR1 and exploits the shuttling mechanism to cross the BBB. To assess non-Aβ driven uptake, B12-8D3, targeting gp120 of HIV-1, was produced and used as a control antibody. The different antibody constructs retained their affinity to Aβ and TfR1 after DFO* conjugation and ^89^Zr radiolabeling (Supplementary Fig. 1 and Supplementary Table 1). The ^89^Zr radiolabeling results of the different antibodies are shown in Supplementary Table 2.

**Table 1:**
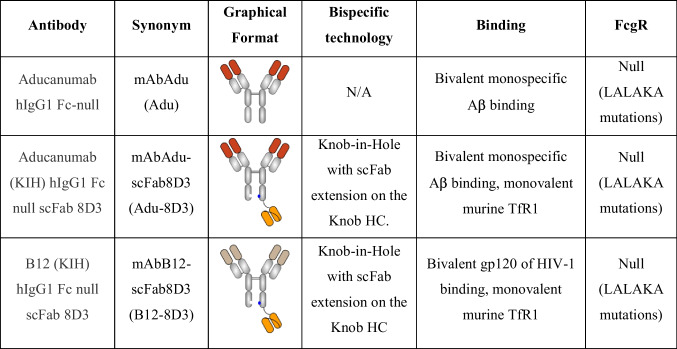
Overview of evaluated antibodies

### Autoradiography and immunofluorescence studies

One brain hemisphere was flash-frozen in isopentane at − 30 °C for 2 min. Tissue cryosections (20 µm) of 4 different brain regions (sagittal cuts) per mouse (*n* = 3 per group) were mounted on glass slides, air-dried, and directly exposed to phosphor screen BAS-IP SR 2040 E (General Electric, Eindhoven, the Netherlands) for 2 weeks. Phosphor screens were imaged using a Typhoon FLA 7000 imager (General Electric, Eindhoven, the Netherlands). The intensity of the signal was quantified using Image Quant software (General Electric, Eindhoven, the Netherlands). After autoradiography exposure, slides were kept at − 20 °C until immunostaining. Before staining, slides were thawed at RT and incubated for 15 min at − 20 °C in acetone, air-dried for 15 min, and blocked for 1 h with 20% normal goat serum (Thermo Fisher, cat. #PCN5000) in phosphate buffer solution (PBS). The injected antibody was detected using Alexa Fluor 647 goat-anti-human IgG (Life Technologies, cat. #A21445) (1:1000 in PBS + 2% goat serum, 1 h, RT, in the dark). After incubation, the slides were washed with PBS + 0.05% Tween-20 3 × 5 min. The Aβ plaques were stained with 0.125% thioflavin-S (Sanbio, cat. #T1892), diluted in distilled water for 8 min, and washed 2 × 3 min with 80% ethanol, 1 × 3 min with absolute ethanol, and 1 × 3 min with distilled water. All stained slides were coverslipped with ProLong™ Gold Antifade Mountant (Invitrogen™, P36930) and imaged with a fluorescence microscope (Zeiss Axio Observer with a Colibri 7 LED light source and an Axiocam 506 monochrome camera). Images shown in the figures are taken from the brain cortex area.

### Preclinical mouse model

Transgenic C57BL/6 J-TG(Thy1-APPSw-Thy1-PSEN1*L166P)21/Jckr mice, designated APP/PS1 TG mice, which carry a transgene insertion for the human amyloid-beta 42, were used. This amyloid beta 42-driven amyloidosis reveals early and robust pathology [34]. The APP/PS1 TG mice and age-matching wild-type (WT) littermates were obtained from Charles Rivers (Germany) at ages 3, 7, and 10 months. The mice were housed and maintained in OptiMice cages under specific pathogen-free conditions. Animal experiments were performed according to the European Community Council Directive (2010/63/EU) for laboratory animal care and the Dutch Law on animal experimentation (“Wet op de dierproeven,” Stb 1985, 336).

### PET imaging studies

PET imaging was performed using small animal NanoPET/CT and NanoPET/MR scanners (Mediso Ltd., Budapest, Hungary) equipped with identical PET components. Mice were anesthetized by inhalation of 1.5–2.5% isoflurane/O_2_ during the scanning period. Mice were positioned on the scanner bed, their respiratory rate was monitored during the entire scan, and anesthesia was adjusted whenever required. Static PET scans were acquired for 60 min at different time points after i.v. injection of [^89^Zr]Zr-DFO*-mAb constructs (5–6 MBq). Reconstruction was performed using a 3-dimensional reconstruction algorithm (Tera-Tomo; Mediso Ltd.) with four iterations and six subsets, resulting in an isotropic 0.4 mm voxel dimension. Radioactivity uptake was calculated as the percentage of the injected dose per gram of tissue (%ID/g) with the decay-corrected amount of injected radiolabeled compound. Images were analyzed and quantified using the VivoQuant software (Invicro, Boston, USA), and regions of interest (ROI) were applied using VivoQuant-integrated brain atlas fitting CT and MRI scans (Supplementary Fig. 7). Data was analyzed using GraphPad Prism 9 (San Diego, CA, USA).

### Biodistribution studies

Biodistribution of [^89^Zr]Zr-DFO*-mAbs was evaluated in APP/PS1 TG mice (Charles River, Germany) and WT littermates of the same breed as controls. The mice’s age was 10 months for the comparative study of the different compounds and the dose escalation study. Mice of age 3, 7, and 10 months were used to evaluate the uptake in mice with different Aβ plaque loads. Every experiment had *n* = 5 mice per group. Mice were i.v. injected into the tail veins with either 1 mg/kg (30 µg) of [^89^Zr]Zr-DFO*-mAb (5–6 MBq) or 100, 200, and 400 µg of [^89^Zr]Zr-DFO*-mAb (5–6 MBq) per mouse in 150–200 µL under 2–4% isoflurane/O_2_ inhalation anesthesia. Blood samples were drawn under 2–4% isoflurane/O_2_ inhalation anesthesia at 2 hours (h), 6 h, 1 day (d), 2 d, 3 d, 4 d, 5 d, 6 d, and 7 d for *n* = 5 mice per group for blood kinetics of [^89^Zr]Zr-DFO*-Adu, [^89^Zr]Zr-DFO*-Adu-8D3, and [^89^Zr]Zr-DFO*-B12-8D3. At d3 or d7 p.i. mice were anesthetized, bled, euthanized, and dissected. Additionally, the mice sacrificed at d7 p.i. were imaged at d1, d3, and d7 p.i. Blood, brains, and organs of interest for all mice were collected and weighed, and the amount of radioactivity in each sample was measured in the Wizard gamma counter (Wallac/PerkinElmer, Waltham, MA, USA). Radioactivity uptake was calculated as the percentage of the injected dose per gram of tissue (%ID/g).

### Statistics

The Grubbs outlier test was used to check and remove outliers, and statistical analysis was performed on the tissue uptake values of the different groups of mice with Welch’s *t*-test. Welch’s *t*-test is a *t*-test for small groups that do not assume that the variances are equal. Both take the normal Gaussian distribution of the values. Two-sided significance levels were calculated, and *p* < 0.05 was statistically significant. All graphs were generated using GraphPad Prism 9 software.

## Results

### TfR1-mediated transcytosis leads to 7-fold higher specific brain uptake

#### Immuno-PET and *ex vivo* biodistribution

**Fig. 1:**
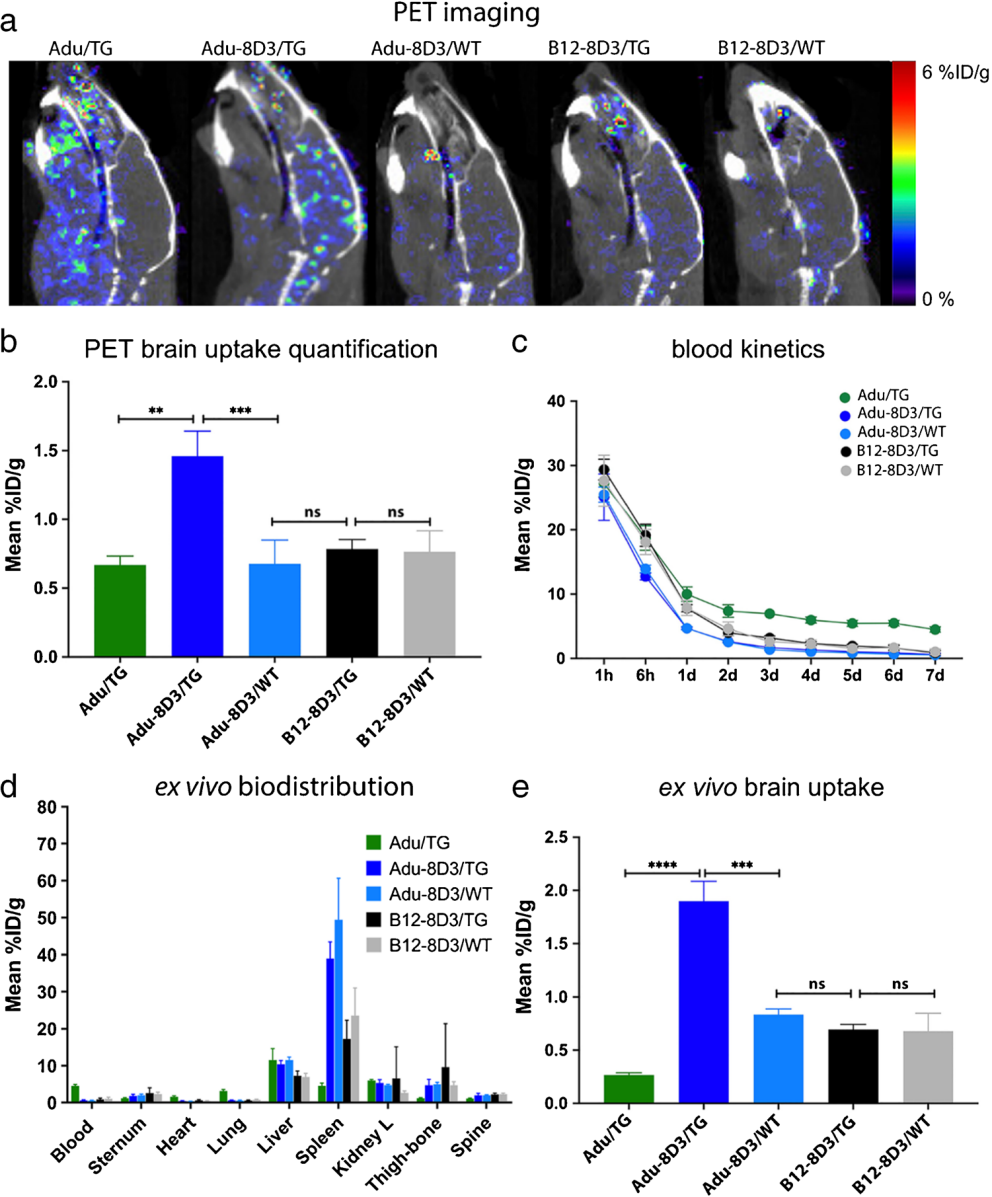
Brain uptake and pharmacokinetics of [^89^Zr]Zr-DFO*-radiolabeled monospecific Adu and bispecific Adu-8D3 and nonbinding B12-8D3 in APP/PS1 TG and WT control mice. **a** Representative sagittal PET images showing brain uptake at d7 p.i.; **b** PET images quantification of brain uptake at d7 p.i.; **c** blood kinetics curves of the different antibodies; **d** *ex vivo* quantification of uptake in selected organs at d7 p.i.; **e** *ex vivo* quantification of brain uptake at d7 p.i.; Uptake is expressed as %ID/g (mean ± SD, *n* = 5 animals per group). **p < 0.01; ***p < 0.001; ****p < 0.0001; ns: nonsignificant, *p* > 0.2, analyzed via *t*-test with Welch correction

**Fig. 2:**
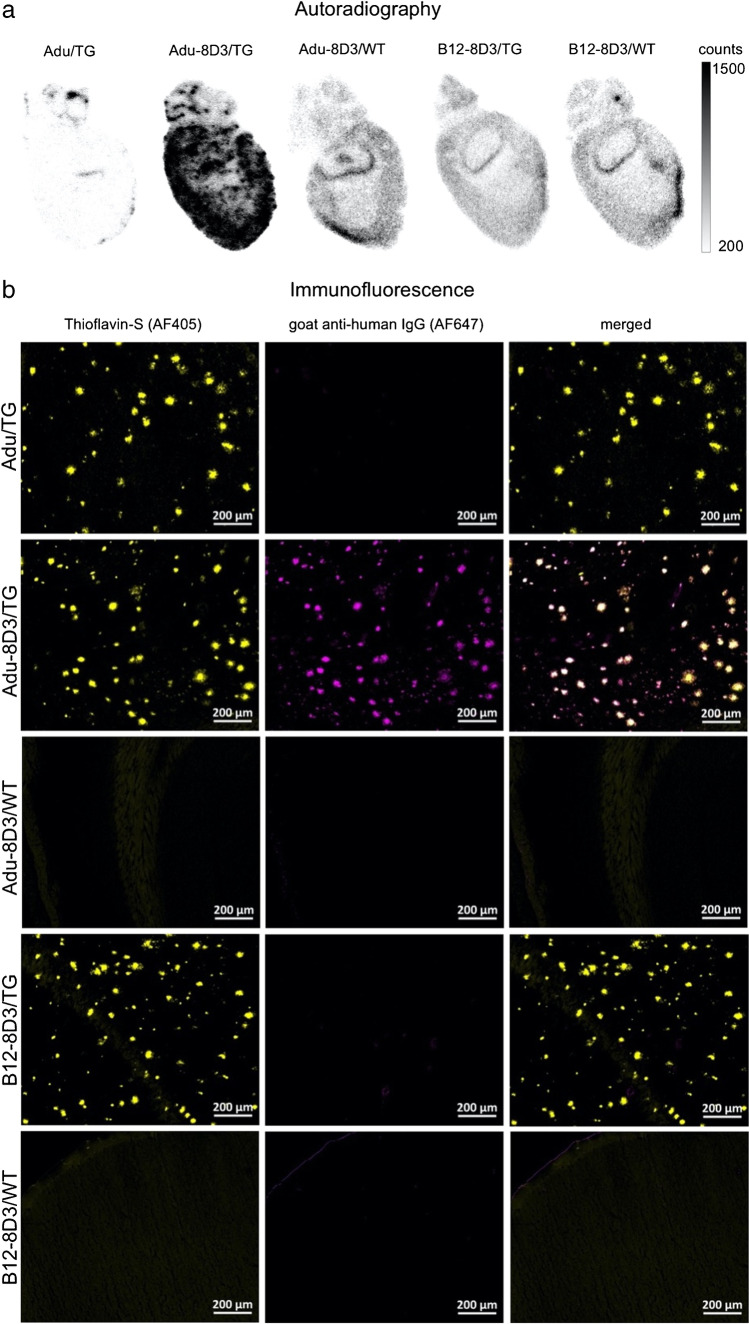
Validation of target engagement of [^89^Zr]Zr-DFO*-radiolabeled monospecific Adu and bispecific Adu-8D3 and B12-8D3 in APP/PS1 TG and WT mice. **a** Representative *ex vivo* autoradiograph of Adu, Adu-8D3, and B12-8D3 in TG and WT mice at d7 p.i.; **b** Immunofluorescence staining of the same autoradiography samples. Thioflavin S (yellow, left lane) and AF647-goat-anti human IgG (purple, middle lane), the merged channels (white, right lane). The immunofluorescence picture appears black in case there is no signal for thioflavin S and no signal for the injected antibody

To evaluate the brain targeting potential of the amyloid-β(Aβ)/TfR1 bispecific Adu-8D3, ^89^Zr-immuno-PET imaging and *ex vivo* biodistribution studies were performed in APP/PS1 TG mice and WT littermates. PET imaging was performed on d1, d3, and d7 post-antibody injection (p.i.) (Supplementary Fig. 2 and Supplementary Table 3), and *ex vivo* biodistribution at d3 and d7 (Supplementary Tables 4 and 6). The best image contrast for all groups was observed at d7. Figure 1 shows the PET brain imaging results (Fig. 1a) and the PET quantification of brain uptake (Fig. 1b) of Adu-8D3, Adu, and B12-8D3 in APP/PS1 TG and WT littermates at d7 p.i. The ^89^Zr-immuno-PET revealed high uptake of Adu-8D3 in the brain of APP/PS1 TG mice, in contrast to the Adu (Fig. 1a). The PET signal shows an Aβ plaque-specific pattern, which is comparable to the pattern of *ex vivo* stainings of the injected Adu-8D3 (Fig. 2). In contrast, the monospecific Adu and the isotype control B12-8D3 showed little uptake in the brain of APP/PS1 TG mice. PET quantification revealed a brain uptake of 1.5 %ID/g for Adu-8D3 and around 0.7 %ID/g for Adu and the control groups (Fig. 1b). The blood pharmacokinetics (Fig. 1c) showed a relatively rapid decrease in blood content for Adu-8D3 over time, with only 0.6%ID/g still present at d7. Isotype control B12-8D3 showed slightly slower pharmacokinetics in APP/PS1 TG and WT littermates compared to Adu-8D3, especially at early time points. However, after d1, the clearance of Adu from the blood slowed, and the curve flattened, reaching 4.6 %ID/g at d7. The faster blood kinetics of the bispecific construct compared to Adu is probably due to the interaction of the 8D3 moiety with TfR1 in the periphery (e.g., spleen), leading to faster blood clearance. The full-body PET images for the different constructs at day 7 p.i. are shown in Supplementary Fig. 8.

An *ex vivo* biodistribution study of the brain and different peripheral organs was performed to confirm the PET imaging results. The *ex vivo* biodistribution at d7 p.i. for the most important organs is shown in Fig. 1d. The Adu-8D3 and B12-8D3 mAbs, in contrast to Adu, showed a very high accumulation in the spleen and a modest uptake in the thigh-bones, spine, and sternum (bone marrow), which can be explained by the level of TfR1 expression in these tissues [35]. The relatively high uptake of all mAbs in the liver can be explained by catabolic processes, a general observation for all intact immunoglobulins, while the uptake in the kidney is related to excretion [36]. The differences in uptake between Adu-8D3 and B12-8D3 in the liver and the spleen can be explained by natural Aβ expression as well as AA-amyloidosis and the formation of fibrillary aggregates of systemic amyloid deposits, mainly in the liver and spleen [37]. At d3, the *ex vivo* biodistribution results showed about tenfold higher uptake in the brain for Adu-8D3 (2.54%ID/g) in APP/PS1 TG compared to Adu (0.26%ID/g) (Supplementary Table 4). The control groups, Adu-8D3 in the WT littermates (1.24%ID/g), B12-8D3 in APP/PS1 TG (1.05%ID/g), and B12-8D3 in WT littermates (1.13%ID/g), showed significantly lower uptake (*p* < 0.001) than Adu-8D3 in APP/PS1 TG. At d7, brain uptake was lower for all constructs except for Adu (Fig. 1e, Supplementary Table 6). The specific uptake of Adu-8D3 was sevenfold higher in comparison to Adu (0.27%ID/g). Adu-8D3 showed 1.85%ID/g, whereas the control groups being Adu-8D3 in WT littermates (0.83%ID/g), B12-8D3 in APP/PS1 TG mice (0.69%ID/g), and B12-8D3 in WT littermates (0.68%ID/g) showed significantly lower uptake. Of note, the level of brain uptake (%ID/g) obtained by *ex vivo* biodistribution differs from the results of the PET imaging using the brain atlas for quantification. The two methods are less congruent, and absolute quantification of tracer uptake in the brain is difficult due to the influence of partial volume effects in PET analysis and the underperformance of the PET reconstruction algorithms with very low organ uptake [31].

#### Validation of target engagement

The localization of the radiolabeled antibodies in the brain was investigated by *ex vivo* autoradiography (Fig. 2a). A very high signal with an Aβ plaques-specific pattern was observed for Adu-8D3 injected in APP/PS1 TG mice. No radioactive signal could be detected for Adu in the brain by autoradiography, and a weak radioactive background signal could be observed for the control groups (Adu-8D3 in WT mice, B12-8D3 in TG mice, and B12-8D3 in WT mice). The same brain tissues were used for immunofluorescence staining of the injected antibodies and Aβ plaques to demonstrate (a) the co-localization of radioactive signal with the injected antibody and (b) target engagement of injected Aβ antibody with Aβ plaques (Fig. 2b). The injected bispecific Adu-8D3 could be detected (purple) and co-localized with the Aβ plaques (yellow), giving a white color signal in the overlay picture. No injected antibody could be detected by immunofluorescence staining for the control groups (Adu in TG mice, Adu-8D3 in WT mice, B12-8D3 in TG mice, and B12-8D3 in WT mice).

### Increasing [^89^Zr]Zr-DFO*-Adu-8D3 protein dose resulted in decreasing Aβ-specific PET signal

**Fig. 3:**
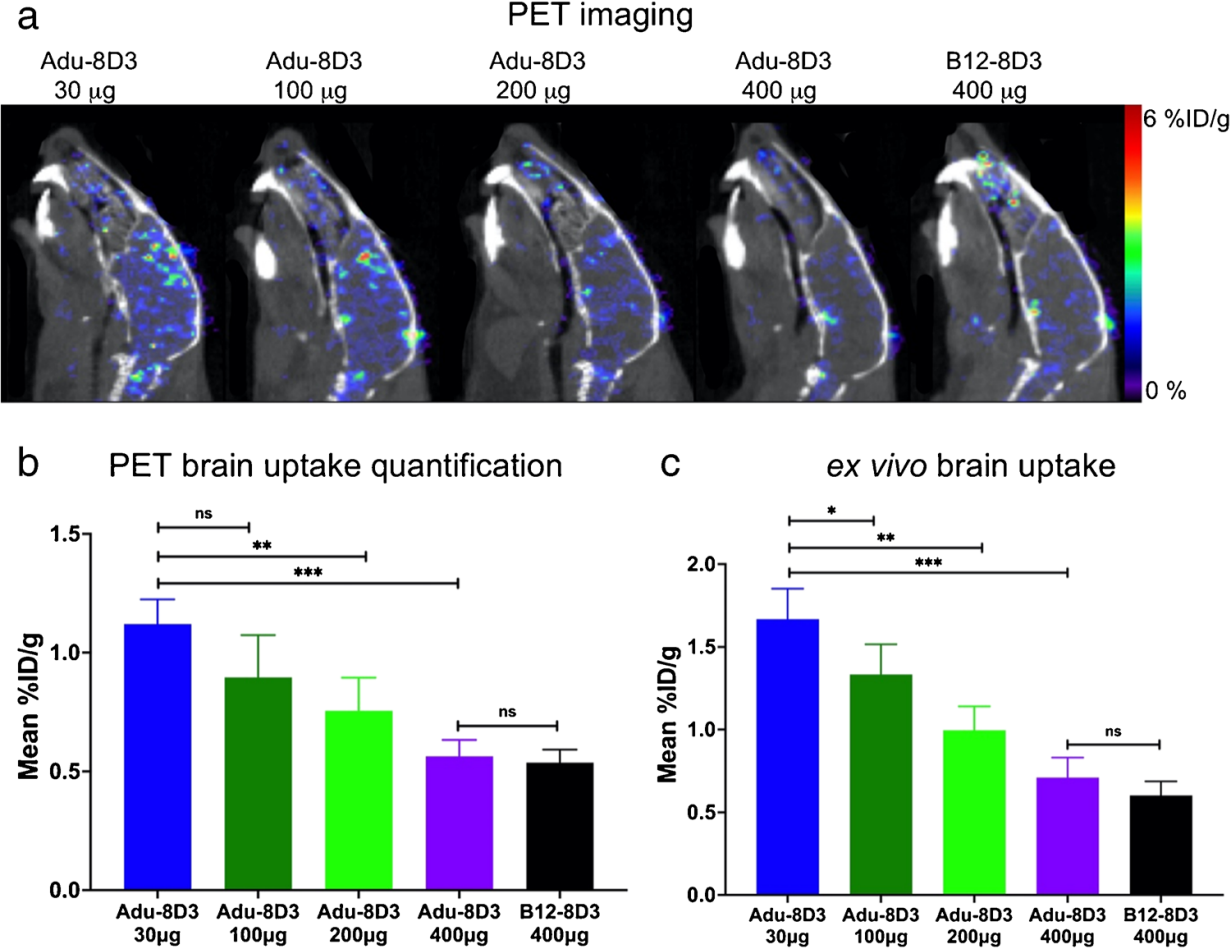
Dose-dependent brain uptake of [^89^Zr]Zr-DFO*-Adu-8D3 in APP/PS1 TG mice. **a** Representative sagittal PET images showing brain uptake of Adu-8D3 in different dose groups and B12-8D3; **b** PET images quantification of brain uptake at d7 p.i; **c** *ex vivo* quantification of brain uptake at d7 p.i. Brain uptake is expressed as %ID/g (mean ± SD, *n* = 5 animals per group). *p < 0.1; **p < 0.01; ***p < 0.001; ns: nonsignificant, *p* > 0.2, analyzed via *t*-test with Welch correction

To investigate the effect of antibody amount on the Aβ-specific brain uptake of Adu-8D3, 4 different antibody doses were evaluated (30, 100, 200, and 400 µg) that contain equivalent radiolabeled fractions (5–6 MBq ^89^Zr). The higher the antibody dose, the larger the fraction of unlabeled antibodies. As a negative control, a group of APP/PS1 TG mice was injected with 400 µg B12-8D3. PET imaging and *ex vivo* biodistribution were performed at d7 p.i. Figure 3 shows the brain PET images (Fig. 3a), the brain uptake quantified by PET imaging (Fig. 3b), and by *ex vivo* biodistribution (Fig. 3c). Aβ-specific detectable brain uptake, defined as the difference in uptake between Adu-8D3 and B12-8D3, was reduced with increasing nonradioactive mAb amount in a dose-dependent way. The brain uptake of 400 µg Adu-8D3 at d7 p.i was comparable to 400 µg B12-8D3 (Fig. 3b and c and Supplementary Table 8). Additionally, a reduction in TfR1-specific uptake in a dose-dependent way could be observed in the periphery of the blood and the spleen (Supplementary Table 9).

**Fig. 4:**
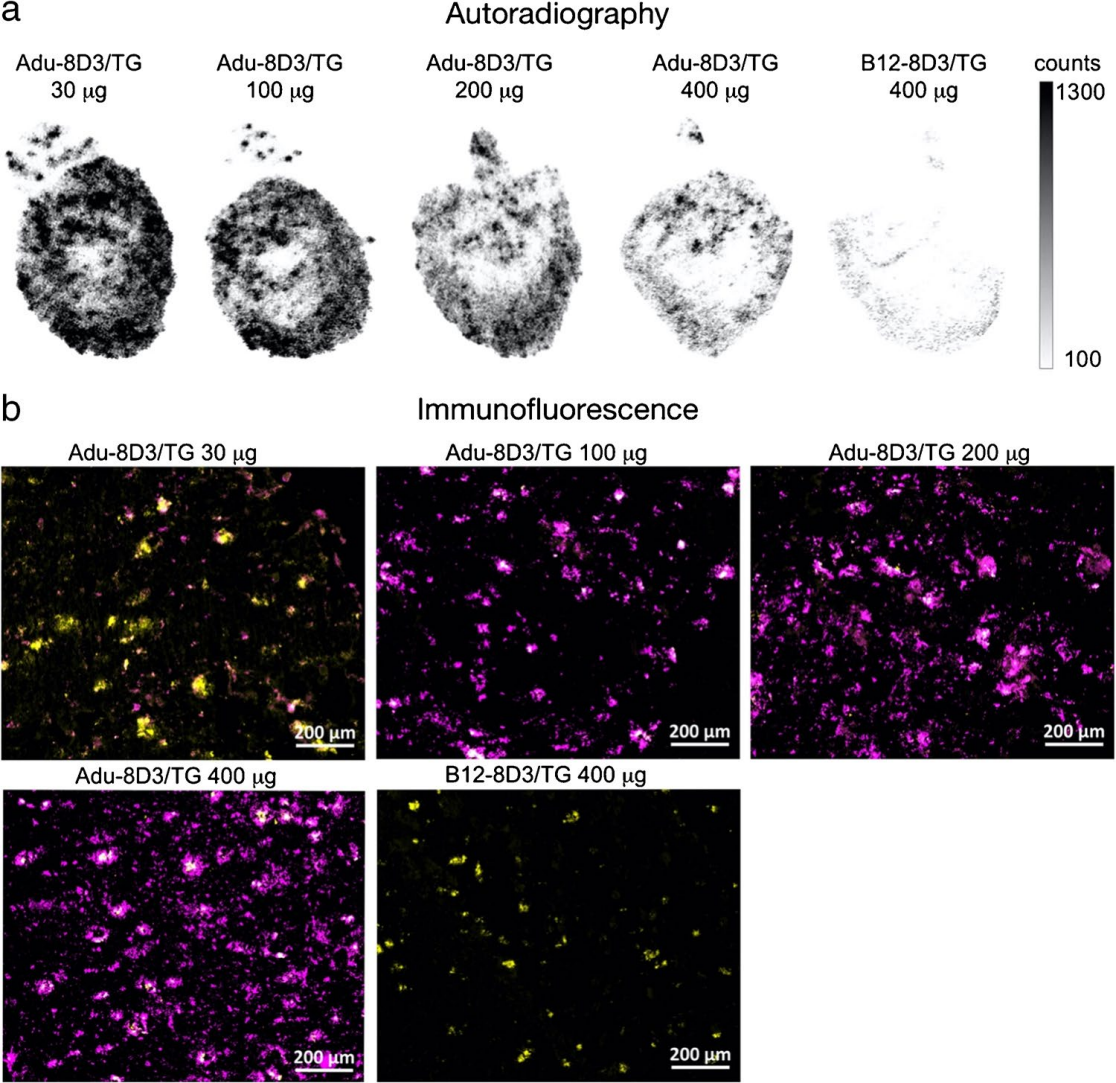
Dose-dependent target engagement of [^89^Zr]Zr-DFO*-radiolabeled bispecific Adu-8D3 and B12-8D3 in APP/PS1 TG mice. **a** Representative *ex vivo* autoradiograph of Adu-8D3 in different dose groups at d7 p.i.; **b** immunofluorescence staining for each group of the very same autoradiography sample shown under (a), thioflavin S (yellow), and AF647-goat-anti human IgG (purple), overlay of the two signals appears in white. Fluorescence microscopy pictures of single channels can be found in Supplementary Fig. 3

In addition, autoradiography showed a decreasing radioactive signal with an increasing mAb dose (Fig. 4a). The immunofluorescence staining of the injected antibody, on the other hand, showed increased staining (Fig. 4b and Supplementary Fig. 3) when more antibody was administered. These results indicate that higher doses of injected antibodies result in more total antibodies in the brain, while the radiolabeled antibody levels are reduced. Quantification of Adu-8D3 amount in the brain normalized to plaque load for the injected amount of Adu-8D3 revealed that with a dose above 200 µg, a trend toward saturation can be seen (Supplementary Fig. 4).

### [^89^Zr]Zr-DFO*-Adu-8D3 can detect low plaque loads in AD mice

**Fig. 5:**
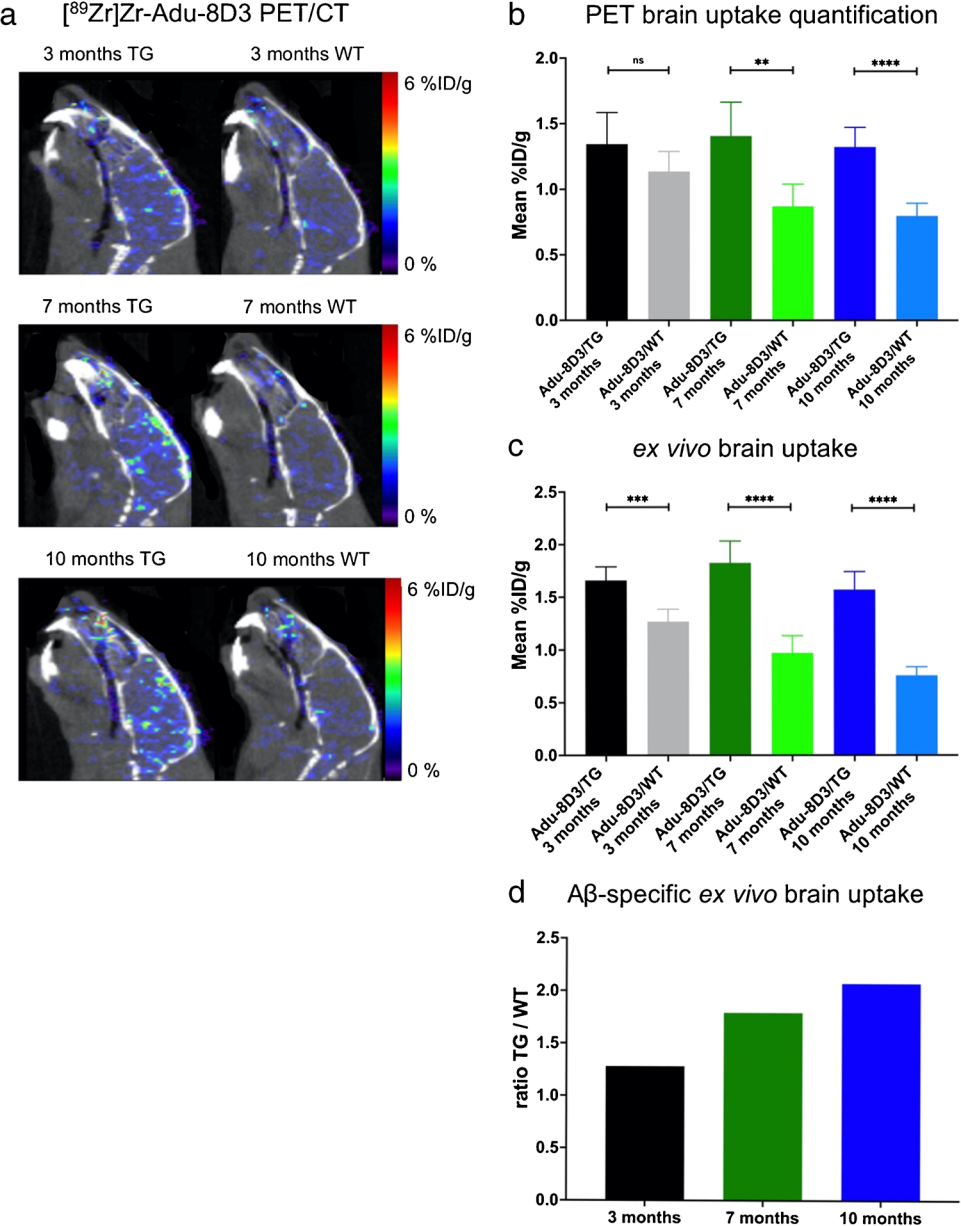
Aβ imaging of different plaque loads with [^89^Zr]Zr-DFO*-Adu-8D3 (1 mg/kg; 30 µg) in APP/PS1 TG and WT littermates at ages of 3, 7, and 10 months. **a** Representative sagittal PET images showing Adu-8D3 uptake in the brain at d7 p.i.; **b** PET images quantification of brain uptake at d7 p.i.; **c** *ex vivo* quantification of brain uptake at d7 p.i.; **d** Aβ specific tracer uptake represented as the ratio of uptake in APP/PS1 TG to uptake in WT littermates of the respective age groups. Brain uptake is expressed as %ID/g (mean ± SD, *n* = 6 animals per group). **p < 0.01; ***p < 0.001; ****p < 0.0001; ns: nonsignificant, *p* > 0.2, analyzed via *t*-test with Welch correction

To evaluate the potential of [^89^Zr]Zr-DFO*-Adu-8D3 as a theranostic in early-stage disease where therapeutic intervention has a higher impact, we examined its use at 1 mg/kg dose for early detection of Aβ plaques. For this study, 3, 7, and 10-month-old APP/PS1 TG mice with different plaque loads (Supplementary Fig. 6) and their age-matched WT littermates were imaged with [^89^Zr]Zr-DFO*-Adu-8D3. For comparison, PET imaging with carbon-11-labeled Pittsburgh compound B ([^11^C]PIB) as the gold standard for amyloid plaque imaging (Supplementary Fig. 9) and quantification by thioflavin-S staining on whole brain slices was performed at the same time points (Supplementary Fig. 6). The brain quantification already showed increased uptake [^89^Zr]Zr-DFO*-Adu-8D3 in 3-month-old APP/PS1 TG mice (1.63%ID/g) compared to WT littermates of the same age (1.27%ID/g) (Fig. 5a). The Aβ-specific signal in the brain was most pronounced at the age of 7 and 10 months when more plaque load was present. PET quantification (Fig. 5b) and *ex vivo* biodistribution (Fig. 5c) showed nearly the same brain uptake for all APP/PS1 TG mice age groups. In contrast, the brain uptake in WT littermates was highest at 3 months and declined with age. Therefore, the uptake ratio in APP/PS1 TG mice divided by the uptake in WT littermates was calculated to determine the specific uptake for Adu-8D3 and showed an increased ratio with the age of the mice (Fig. 5d).

**Fig. 6:**
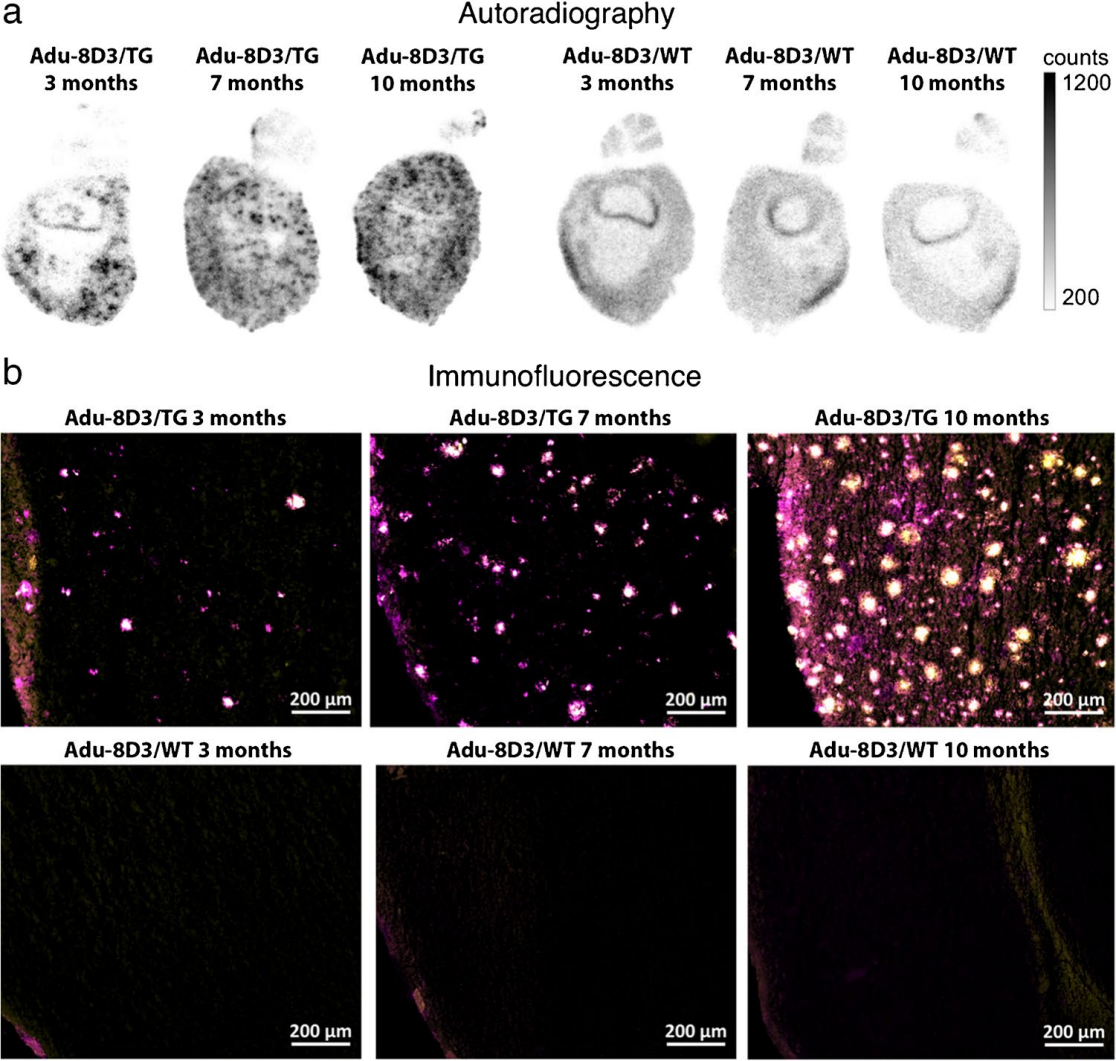
Plaque load-dependent target engagement of [^89^Zr]Zr-DFO*-Adu-8D3 in APP/PS1 TG and WT mice. **a** Representative *ex vivo* autoradiograph of Adu-8D3 in the different age groups at d7 p.i.; **b** immunofluorescence staining of the same autoradiography sample shown for each group. Thioflavin S (yellow) and AF647-goat-anti human IgG (purple) are the pictures of the merged channels, an overlay of the two signals appears in white. Fluorescence microscopy pictures of single channels can be found in Supplementary Fig. 5

*Ex vivo* autoradiography was also performed on the brain at d7 p.i. (Fig. 6a). [^89^Zr]Zr-DFO*-Adu-8D3 showed a plaque-specific pattern in the APP/PS1 TG mice, which co-localized with the Aβ plaques stained by immunofluorescence (Fig. 6b). The autoradiography images show an increased signal with the age of the mice, which correlates well with the levels of amyloid pathology. Furthermore, autoradiography in WT littermates showed a higher antibody signal in young mice compared to older mice, which correlates with the PET imaging observations (Fig. 6a). Immunostaining for Aβ showed an increased level of amyloid pathology with the age of mice. A similar increase in staining was observed for the injected antibody, which correlates with the autoradiography and PET imaging findings.

## Discussion and conclusion

Amyloid-PET imaging was a major contributor to the approval of aducanumab, as it was applied to screen patients for proof of Aβ positivity and thus eligibility for treatment with aducanumab. Furthermore, [^18^F]florbetapir PET imaging was used to demonstrate the clearance of the Aβ plaques during/after therapy with aducanumab in a dose-dependent manner [10]. Thus, the use of small-molecule amyloid PET in clinical trials has contributed to the FDA approval of aducanumab, and the continued use of this biomarker in AD clinical trials has reinforced the use of PET imaging in drug development [38]. It is believed that amyloid PET imaging can offer tremendous and unconditional support in early diagnosis, patient selection, monitoring of the disease, and assessing the therapeutic effect of new drugs against AD [39]. However, monitoring the drug itself and its brain delivery over the BBB is still lacking, slowing the development of effective immunotherapies for neurological diseases. A theranostic approach using [^89^Zr]Zr-DFO*-immuno-PET is a valuable quantitative tool capable providing quantitative information on the amount of antibody that enters the brain, target engagement, and mAb kinetics.

More importantly, ^89^Zr-DFO*-immuno-PET has high potential in supporting the development and evaluation of new bispecific Aβ-specific antibodies designed for improved brain delivery by providing quantitative data on brain exposure. In this study, we showed that Adu-8D3 resulted in a sevenfold higher brain uptake than Adu (Fig. 1), demonstrating the improved brain uptake of Adu-8D3 by exploiting brain shuttling mechanisms via the TfR1. Targeting TfR1 with the 8D3 clone is a viable option to improve the delivery of biologics to the brain in preclinical mouse models. Furthermore, multiple studies using RMT mechanisms demonstrated that a longer half-life (reduced clearance) and prolonged circulation of brain shuttle constructs increase their interactions with the receptor at the BBB, resulting in higher brain drug delivery [17].

When targeting ubiquitously expressed receptors like TfR, optimal dosing and dose scheduling in humans is very important; ^89^Zr-DFO*-immuno-PET imaging can provide guidance in this assessment [40]. The dose escalation experiment of Adu-8D3 with the dose range we explored (30–400 µg) showed no apparent sink effect due to TfR1 expression in peripheral organs. It is possible that a sink effect would occur at a lower dose than 30 µg; however, this could not be explored due to the limitation of the antibody labeling with higher molar activity. By adding different amounts of non-radioactive mAb to the radiolabeled formulation, the binding to the TfR1 receptor gets blocked in the periphery and at the BBB, resulting in reduced detectable brain uptake of the radiolabeled Adu-8D3 (Fig. 3 and Supplementary Tables 8 and 9). However, blocking of Aβ in the brain cannot be excluded, and the reduced brain uptake with the higher doses of nonradioactive Adu-8D3 is most likely a contribution of the blocking of TfR1 as well as Aβ binding. Regarding the best dosage for imaging, low doses with high molar activity are needed to generate PET images with good quality and high contrast. Additionally, our dose escalation experiment showed a correlation between injected dose and the percentage antibody entering the brain (Fig. 4), highlighting the importance of proper dosing for optimal brain delivery of therapeutics with a brain shuttling mechanism. ^89^Zr-DFO*-immuno-PET can support the development of therapeutics and can additionally monitor their efficiency by imaging surrogate endpoints.

Due to the improved brain uptake of Adu-8D3 achieved by exploiting the TfR1 shuttle mechanism, the therapeutic use of Adu-8D3 in a lower dose than aducanumab is conceivable. To use the new bispecific mAb therapeutically in humans, the 8D3 scFab directed against murine TfR1 must be replaced by a human TfR1 binding moiety. To our knowledge, only one bispecific anti-Aβ antibody (gantenerumab) coupled to a scFab specific for TfR1 has completed a phase I trial (NCT04023994), and preliminary data suggest improved efficacy. This highlights the opportunity for developing antibody conjugates exploiting the TfR1 shuttle mechanism to improve brain uptake and facilitate the use of lower antibody doses in the clinic. ^89^Zr-immuno-PET, combined with DFO* as a chelator, can be a powerful tool to derisk the development of novel brain-shuttling antibody constructs and biologicals for neurological disease. However, ^89^Zr-immuno-PET for brain imaging with bispecific antibodies is still in its early stages, and many questions are still not explored. (1) How stable are the ^89^Zr conjugates in the brain? What is the fate of the free ^89^Zr? and how is it cleared from the brain? (2) Is there any adverse effect of the ^89^Zr accumulation in the brain? (3) Does the chelator-to-antibody ratio affect brain shuttling and kinetics? (4) What is the ideal plasma half-life of conjugates for optimal brain exposure? Is there any amyloid-related imaging abnormalities (ARIA) event related to the brain shuttling bispecific amyloid-β antibodies?

With this in mind, early AD diagnosis and subsequent treatment intervention in the presymptomatic stage of the disease will likely slow down or halt the progression. In addition, ^89^Zr-DFO*-immuno-PET imaging and theranostics can be powerful tools for providing patients with specific and personalized treatment.

## Supplementary Information

Below is the link to the electronic supplementary material.

Supplementary file1 (DOCX 5076 KB)

## Data Availability

The datasets generated during and/or analyzed during the current study are available from the corresponding author upon reasonable request.

## Abbreviations

ARIA: Amyloid-related imaging abnormalities
AR: Autoradiography
mAb: Monoclonal antibody
Zr: Zirconium
DFO*-NCS: P-NCS-Bz-DFO*
DFO-NCS: P-NCS-Bz-DFO
*H*: Hour
*S*: Second
Min: Minute
*W*: Week
*D*: Day
C: Carbon
PIB: Pittsburgh compound-B
HPLC: High-pressure liquid chromatography
ELISA: Enzyme-linked immunosorbent assay
FACS: Fluorescence-activated cell sorting
AD: Alzheimer’s disease
A $$\upbeta$$: Amyloid-$$\upbeta$$
TG: Transgenic
WT: Wild-type
CDR: Complementarity-determining region
IF: Immunofluorescence
F: Fluor
