# Supplementary material for: Application of 89Zr-DFO*-immuno-PET to assess improved target engagement of a bispecific anti-amyloid-ß monoclonal antibody
Source: Eur J Nucl Med Mol Imaging. 2023 Jan 13;50(5):1306–17. doi: 10.1007/s00259-023-06109-3 (PMC10027647; doi:10.1007/s00259-023-06109-3)
Supplement: Supplementary file 1 — Supplementary file1 (DOCX 5076 KB) [file 259_2023_6109_MOESM1_ESM.docx]

**Supplementary Information**

**Materials and methods**

**General materials**

Starting reagents and solvents were obtained from Sigma-Aldrich® (dimethylsulfoxide (DMSO), Na_2_CO_3_, oxalic acid, Bovine Serum Albumin (BSA), ethanol), Merck Millipore (sucrose, Tween 20® - pharmaceutical grade), Brunschwig Chemie (Phosphate Buffer Salt (PBS)), Biosolve (acetone) or Invitrogen (1M HEPES). In addition, materials were obtained from Greiner Bio-One B.V. (15 ml and 50 ml falcons), GE healthcare (PD10 column), Eppendorf (1.5 ml tubes), ^89^Zr in 1 mol/L oxalic acid was obtained from PerkinElmer (Boston, Massachusetts, USA). Water was distilled and deionized using a Milli-Q® water filtration system (Millipore Sigma, Burlington, Massachusetts, USA). DFO*-NCS was synthesized by Mercachem B.V. (Nijmegen, The Netherlands).

**Antibody design**

All antibodies are built on the human IgG1 framework and hold Fc-null mutations K322A, L234A and L235A to abolish binding to FcgR. Bispecific mAbs with monospecific murine TfR1 binding (scFab 8D3, supplementary Table 1 showing EC50 values for binding to native TfR1) were generated using the Knobs-in-Hole (KIH) technology. On the Knob heavy chain (HC), a 6xG4S linker followed by the 8D3 scFab extension was engineered at the C-terminal. The Hole HC has an average Fc length.

**Cell culture**

Synthetic genes of the heavy chain (HC) and light chain (LC) optimized with human codon composition were sub-cloned into the pTT5 vector for transient expression. Transfection of HC and LC expression vectors was performed in HEK293 6E cells using PEIpro (Polyplus) as a transfection reagent. The HEK293 6E expression system, including the pTT5 vector, was licensed from the National Research Council of Canada (NRCC). Transfected cells were cultured until the viability had dropped to around 50 %, and culture media was harvested by centrifugation and sterile filtration and kept cold (4°C) until purification.

**Antibody purification**

Antibodies in the cell culture harvest were purified by capturing using HiTrap protein G (Cytiva), followed by washing with PBS and elution with 0.1 M Glycine pH 2.7. After dialysis against 20 mM Tris pH 7.5, the sample was passed through a Q-sepharose column (Cytiva) and equilibrated with the same buffer. The flow-through was concentrated to <5 ml and separated using HiLoad® 16/600 Superdex® (Cytiva) in PBS. Fractions were analyzed by SDS-PAGE, SEC, and LC-MS. Selection for pooling was made to minimize aggregates, incorrectly paired molecules, and free LC.

**DFO*-NCS modification and ^89^Zr labelling**

[^89^Zr]Zr-DFO*-NCS-mAbs were produced as described previously (Vugts, D. J. *et al.* *Eur. J. Nucl. Med. Mol. Imaging* **44**, 286–295 (2017)) with slight modifications concerning mainly the conjugation time (2h instead of 30 min) and the molar excess of DFO*-NCS (10x instead of 3x). Briefly, stock concentrations of mAb were diluted to 5 mg/mL with 0.9% NaCl, the pH adjusted to pH 8.9-9.1 with 0.1 M Na_2_CO_3,_ and reacted with 10 equivalents of DFO*-NCS (5 mM, dissolved in DMSO) at 37 °C for 2h. At the end of incubation, the reaction mixture was applied on a PD-10 column (GE Healthcare Life Sciences). DFO*-NCS-mAb was collected in 2 mL of 50 mM sodium acetate/200 mM sucrose, pH 5.4-5.6 (formulation buffer). The concentration of DFO*-NCS-mAb was determined using the non-conjugated mAb as a reference standard with HPLC (see hereafter for the description on QC). Radiolabelling of DFO*-NCS-mAb with ^89^Zr was performed following the same conditions published before (Vugts, D. J. *et al.* *Eur. J. Nucl. Med. Mol. Imaging* **44**, 286–295 (2017)). Typically, for *in vivo* studies, 200 μL ^89^Zr (∼60 MBq) in 1 M oxalic acid solution and 90 μL 2M Na_2_CO_3_ were combined and reacted for 3 min. Subsequently, the modified mAb (∼1.5 mg) diluted in 0.5 M HEPES buffer (pH 7.0) up to 1.71 mL was added to the reaction mixture and incubated for 2 h at room temperature (RT) under constant agitation. The reaction mixture was then applied to a PD-10 column, and [^89^Zr]Zr-DFO*-NCS-mAb was collected in 2 mL of formulation buffer (50 mM sodium acetate/200 mM sucrose + 0.01% Tween-20, pH 5.4-5.6). The radiolabelling yield for all labelling reactions was between 82 and 90%, and the concentration of [^89^Zr]Zr-DFO*-mAbs between 0.3 and 0.6 mg/mL as determined by HPLC analysis. Unlabelled mAb was further added for the formulation of the reaction mixture to a total mAb dose of 30 (1 mg/kg), 100, 200, or 400 μg per mouse. Possible impairment of the binding to Aβ after modification with DFO*-NCS and ^89^Zr radiolabelling was excluded by ELISA analysis (supplementary Fig. 1). An impairment of the binding to TfR1 was also tested via FACS analysis and excluded (Supplementary Table 1).

**Quality controls of radioimmunoconjugates: radiochemical purity, concentration, and integrity (SE-HPLC, spin filter) of conjugates**

Radioimmunoconjugates were checked for mAb integrity and chelator-to-mAb ratio as extensively described and illustrated by Wünsche et al. (In press, Theranostics 10.7150/thno.73509) In addition, their radiochemical purity was assessed by size-exclusion high-performance liquid chromatography (SE-HPLC) and spin filter analysis as described before (Wünsche *et al.*). In short, a Shimadzu HPLC system was equipped with a Superdex® 200 Increase 10/300 GL (30 cm × 10 mm, 8.6 μm) size exclusion column (GE Healthcare Life Sciences) and a guard column using 0.05 M phosphate buffer/0.15 M NaCl/0.01 M NaN_3_ (pH 6.7) as mobile phase with a run time of 40 min at 0.75 mL/min. The radioactivity was monitored with an inline NaI(Tl) radioactivity detector (Raytest Sockett). The radioimmunoconjugates eluted at approximately 15 min and ^89^Zr/^89^Zr-chelator at around 27 min. Antibody concentration and integrity were assessed on the same SE-HPLC system using the areas under the curve on the UV channel at 280 nm. The concentration was determined against a calibration curve of the non-conjugated compound.

The radiochemical purity of each radioimmunoconjugate was also assessed by spin filter analysis following a described procedure (Vugts, D. J. *et al.* *Eur. J. Nucl. Med. Mol. Imaging* **44**, 286–295 (2017)). The wash buffer consisted of a formulation buffer containing 5% DMSO. Four microliters of product diluted to 100 µL with wash buffer were pipetted onto a 30 kDa cut-off spin filter (Microcon-30kDa centrifugal filter unit with Ultracel YM-30 membrane, regenerated cellulose, Merck Millipore), which was subsequently centrifuged at 14000 rpm for 7 min (Eppendorf 5430). The filter was then washed with 100 µL of the wash buffer and spun again for 7 min at 14000 rpm before being washed a second time with 100 µL buffer and spun down again at the same settings. Subsequently, the filter and combined filtrate were counted separately in a gamma counter (Wallac lkd 12a2 pharmacia), and radiochemical purity was determined by calculating the ratio of counts on the filter (with background subtracted) to the total number of counts (filtrate plus the counts on the filter (with background subtracted)).

**[^11^C]PIB synthesis**

Carbon-11 (^11^C) was produced by irradiation of nitrogen through the 14N(p,α)11C nuclear reaction. The formed ^11^C reacted with the oxygen present in the target to form [11C]CO2. The target gas containing the [11C]CO2 was transported with helium and concentrated on a liquid nitrogen-cooled trap. When heating the trap, the [11C]CO2 was released and passed through a solution of LiAlH4 in THF, where it was reduced to [11C]CH3OLi. After evaporating THF, HI in water was added, and the [11C]CH3OLi complex was converted to [11C]methyl iodide ([11C]CH3I). The [11C]CH3I was then distilled and dried over a NaOH/sicapent column, after which it was used in a subsequent reaction. The dry [11C]CH3I was passed through a graphpac/silver triflate column at 200°C, where it was converted to [11C]methyl trifluoromethanesulfonate ([11C]methyl triflate). Next, the [11C]methyl triflate was distilled and passed through a solution of PIB precursor in acetone. This mixture was heated at 60°C for 1 min, then cooled to 20°C and quenched with 1 mL mobile phase. The crude product was purified by semi-preparative HPLC. After diluting the collected fraction with 60 mL water for injection, the product was trapped on a tC18 solid-phase extraction (SPE) cartridge. The HPLC mobile phase was removed by washing the cartridge with 25% ethanol in water for injection (20 mL). The product was then eluted with ethanol, reformulated in saline, sterile filtered, and dispensed.

**ELISA**

To evaluate the influence of modification and radiolabelling of the bispecific anti-amyloid-β antibody Adu-8D3 on the binding to the target, ELISA was performed as *in vitro* control for all radiolabelled constructs. [^89^Zr]Zr-DFO*-B12-8D3 served as negative control. Costar® Assay 96 well polystyrene plates (flat bottom, half area, high binding) were coated with 50 µL/well of 100 ng/mL Var24-peptide in 0.1 M borate buffer (pH 11) at 4°C overnight. After disposing, blocking was performed using 150 µL/well 2% BSA in PBS, shaking the plate at RT and 600 rpm for 2 h. After disposing of the blocking solution, incubation was performed in duplicates with graded antibody concentrations reaching from 0 – 250 ng/mL in incubation buffer (0.1% BSA in PBS + 0.05% Tween20) at RT and 600 rpm for 1 h (11 points horizontally, 1:2 dilutions, 50 µL/well). Subsequently, the wells were washed three times with 0.1% Tween20 in PBS and incubated with 50 µL/well of goat anti-human IgG (H+L) cross-absorbed-HRP secondary antibody (Invitrogen, cat. #31412, 0.8 µg/mL in incubation buffer) at RT and 600 rpm for 1 h. After incubation, the wells were washed 4x with 0.1% Tween20 in PBS and 1x with dH2O before adding 50 µL/well of TMB substrate for 5 to 10 min without shaking in the dark. The enzymatic reaction was stopped with 50 µL/well of 0.5 M HCl solution and absorbance at 450 nm was measured immediately with a microplate reader (TriStar2 multimode reader LB 942, Berthold Technologies). The absorbance of 0 ng/mL primary antibody was used as background value and subtracted from the other values. Values are given in relative absorbance by dividing all values by the highest absorbance value of each row. In case of the negative control, the highest average absorbance of the unmodified Adu-8D3 was used.

**Affinity of the different antibody constructs to Amyloid-β and mTfR1**

**Flow cytometry**

CHO-S cells stably transfected with murine TfR1 (mTfR1) and mock transfected CHO-S cells were cultured in ExpiCHO Expression Medium (Gibco, cat# A29100-01) with 1% anti-clumping agent (Gibco, cat# 0010057AE) at a cell concentration between 1x10^5 and 2x10^6 viable cells/mL in shaker flasks at 37°C, 5% CO_2_. The mTfR1 high expressing cells were supplemented with selection pressure (12 mg/mL Puromycin dihydrochloride (Merck, cat# P9620-10ML)). To prepare the mTfR1 transfected and mock transfected CHO-S cells for FACS, cells were harvested, washed 3 times with cold PBS (spin 300 x g for 5 mins), counted (viable cell count, Cedex Hires) and adjusted to 5x10^6 cells/mL in cold PBS. 100 μL of cells were transferred into a 96-well-multi dish (U bottom) plate (0.5x10^6 cells/well), spinned down for 5 min at 400 x g at 4 °C and the supernatant was discarded. The cells were stained with Live/dead cell stain (L34963, 405nm, Invitrogen). Therefore, a mastermix of 0.1 µL dye in 100 µL PBS w/o Mg^2+^ and Ca^2+^ per sample was prepared. The cells were incubated on ice for 15 min in the dark and washed 3 times by adding a total of 180 μL cold FACS buffer (250 μL PBS w/o Ca^2+^ and Mg^2+^ + 2 mM EDTA (1 mL 0.5 M), 2% normal goat sérum) per well and centrifuged for 5 min with 350 x g at 4 °C. The cells were blocked by adding 50 µL 10% normal goat serum containing buffer/well (1x PBS w/o Ca^2+^ and Mg^2+^ + 2 mM EDTA (60 µL 0.5M), 10 % normal goat serum), mixed well and incubated on ice in the dark for 15 min. After that, the plate was spinned down and the supernatant was discarded. The non-modified and with DFO* modified antibodies (Adu, Adu-8D3, B12-8D3 produced by H. Lundbeck A/S) were added in a total volume of 85 μL with a concentration of 1nM/2x10^5 cells, incubated for 20 min on ice in the dark, 3 times washed as described above with FACS buffer. Secondary antibody, goat anti-human IgG AF647 (Jackson, cat. # 109-605-008) diluted 1:400 in FACS buffer was added to the wells, incubated for 20 min on ice in the dark and washed 3 times with FACS buffer. The cells were fixed with 100 μL of 4% PFA (BD Fixation buffer, cat# 554655) and incubated for 15 min on ice in the dark. Cells were washed 3 times with FACS buffer and resuspended in 180 μL FACS analysis buffer (1xPBS + 2mM EDTA, 1% BSA (0.1g/10 mL) (IgG/protease free, Jackson # 001-000-162, lot# 138456)). Samples were kept on ice in the dark until FACS analysis using a NovoCyte Quanteon. Cells were gated for live, single cells and the mean fluorescence intensity (MFI AF647) and the percentage of binding was determined for each mAb and modified mAb in comparison to unspecific binding of the secondary antibody conjugated with AF647 (FlowJo 10 software).

**Table 1:** Flow cytometry analysis to validate retained binding to mTfR1 after DFO*-conjugation. EC50 values were calculated by using the mean fluorescence intensity (MFI) values and the following formula [Agonist] vs. [response] – [Variable slope] ((four parameters), GraphPad Prism 9.3.1).

| **mAb** | **MFI (AF647)** | **%-binding** | **EC50 (nM)** |
| --- | --- | --- | --- |
| Adu | 189 | 0 | NA |
| Adu-8D3 | 54413 | 97 | 0.45 |
| DFO*-Adu-8D3 | 42824 | 100 |  |
| B12-8D3 | 23955 | 97 | 0.54 |
| DFO*-B12-8D3 | 24897 | 97 |  |


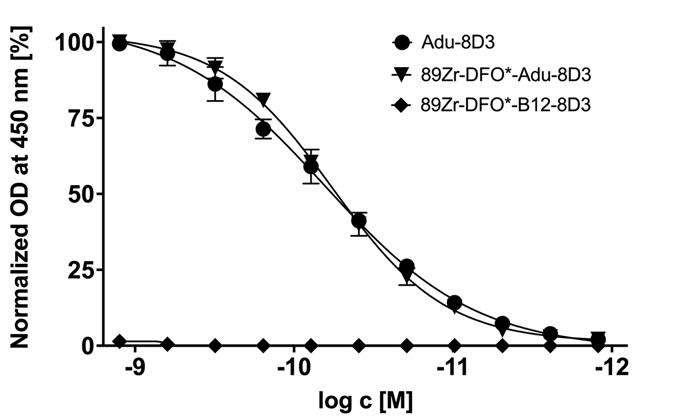


**Figure 1:** Amyloid-β (Aβ) peptide ELISA to determine unimpaired binding to Aβ after DFO*-conjugation and ^89^Zr radiolabeling. Binding analysis of Adu-8D3 and the modified constructs ^89^Zr-DFO*-Adu-8D3 and ^89^Zr-DFO*-B12-8D3.

**Quality controls of radioimmunoconjugates**

**Table 2:** Radiolabelling results of the different antibodies.

| **Antibody** | **Radiochemical yield (%)** | **Radiochemical purity (%)** | **Specific activity (MBq µg^-1^)*** |
| --- | --- | --- | --- |
| Adu | 82 | ≥97 | 0.16-0.17 |
| Adu-8D3 | 79-88 | ≥97 | 0.17-0.21 |
| B12-8D3 | 80-87 | ≥97 | 0.16-0.20 |

*Calculated for 30 µg (1mg/kg) injected dose.

**PET imaging at different time points**


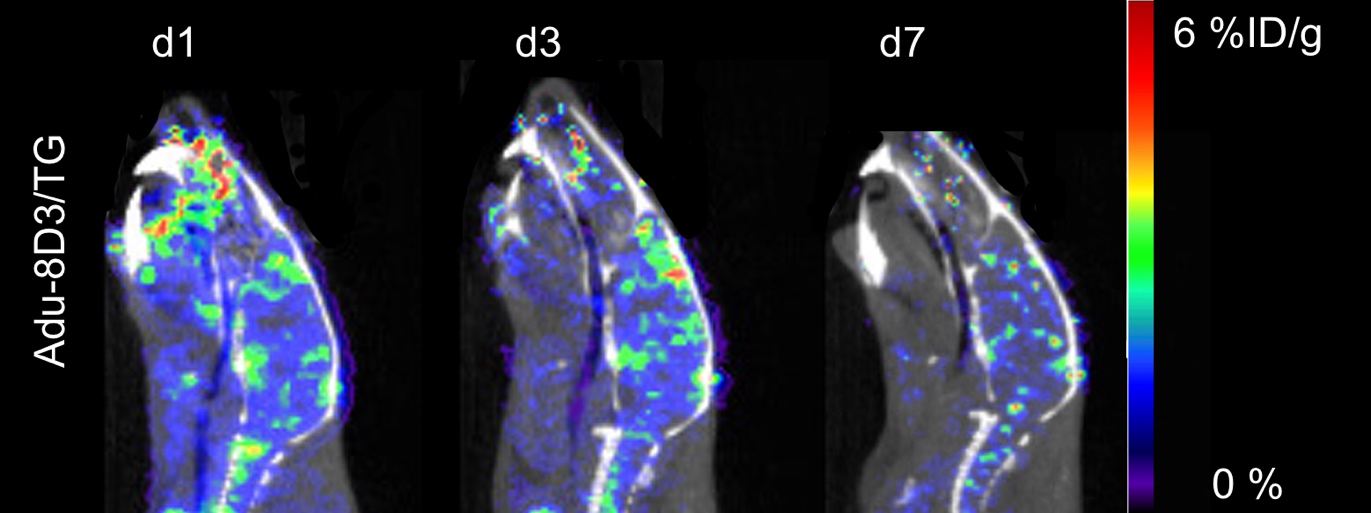


**Figure 2:** PET/CT imaging of injected [^89^Zr]Zr-DFO*-Adu-8D3 (1 mg/kg) at 1, 3 and 7 days p.i. in APP/PS1 transgenic mice (TG). A representative image of one mice imaged at different time points is shown.

**Table 3:** PET quantification of brain uptake of [^89^Zr]Zr-DFO*-mAb conjugates in 10 months old APP/PS1 transgenic mice (TG) or 10 months old WT littermates, d1, d3, d7 after administration of 1 mg/kg of conjugate. Results are expressed as mean (%ID/g) ± sd (n=5 mice per group).

| Group | d1 | d3 | d7 |
| --- | --- | --- | --- |
| Adu/TG | 1 ± 0.08 | 0.86 ± 0.09 | 0.67 ± 0.06 |
| Adu-8D3/TG | 2.09 ± 0.1 | 1.88 ± 0.1 | 1.46 ± 0.1 |
| Adu-8D3/WT | 1.88 ± 0.16 | 1.3 ± 0.13 | 0.68 ± 0.17 |
| B12-8D3/TG | 1.9 ± 0.13 | 1.31 ± 0.11 | 0.78 ± 0.07 |
| B12-8D3/WT | 1.94 ± 0.25 | 1.3 ± 0.25 | 0.76 ± 0.15 |

**Biodistribution studies**

**Table 4:** Biodistribution of [^89^Zr]Zr-DFO*-mAb conjugates in 10 months old APP/PS1 transgenic mice (TG) or 10 months old WT littermates at d3 after administration of 1 mg/kg of conjugate. Results are expressed as mean (%ID/g) ± sd (n=5 mice per group).

| **Organ** | **Adu/TG** | **Adu-8D3/TG** | **Adu-8D3/WT** | **B12-8D3/TG** | **B12-8D3/WT** |
| --- | --- | --- | --- | --- | --- |
| Blood | 6.76 ± 0.57 | 1.93 ± 0.13 | 1.32 ± 0.11 | 2.72 ± 0.18 | 2.74 ± 0.26 |
| Urine | 1.98 ± 0.29 | 2.23 ± 0.58 | 1.97 ± 1.55 | 2.65 ± 0.36 | 3.18 ± 1.37 |
| Skin | 1.60 ± 0.43 | 0.74 ± 0.07 | 0.55 ± 0.14 | 0.73 ± 0.18 | 0.69 ± 0.08 |
| Bladder | 4.55 ± 0.47 | 2.01 ± 0.20 | 1.59 ± 0.23 | 1.96 ± 0.15 | 1.9 ± 0.28 |
| Sternum | 1.44 ± 0.13 | 2.47 ± 0.33 | 2.33 ± 0.38 | 2.45 ± 0.64 | 3.13 ± 0.67 |
| Heart | 2.47 ± 0.11 | 0.98 ± 0.06 | 0.7 ± 0.09 | 1.02 ± 0.08 | 1.01 ± 0.12 |
| Lung | 4.38 ± 0.54 | 1.55 ± 0.06 | 1.23 ± 0.1 | 1.50 ± 0.24 | 1.65 ± 0.18 |
| Liver | 12.32 ± 2.20 | 12.97 ± 1.71 | 11.82 ± 1.42 | 6.64 ± 0.91 | 6.41 ± 0.49 |
| Pancreas | 1.32 ± 0.13 | 0.55 ± 0.12 | 2.52 ± 4.62 | 0.51 ± 0.1 | 0.37 ± 0.04 |
| Spleen | 4.83 ± 0.95 | 47.49 ± 8.22 | 42.16 ± 6.43 | 20.69 ± 1.33 | 19.3 ± 3.75 |
| Kidney L | 7.12 ± 0.37 | 7.24 ± 0.54 | 9.35 ± 5.45 | 3.43 ± 0.18 | 3.39 ± 0.24 |
| Muscle | 0.67 ± 0.06 | 0.23 ± 0.01 | 0.26 ± 0.03 | 0.32 ± 0.04 | 0.35 ± 0.02 |
| Thigh-bone | 1.37 ± 0.10 | 6.79 ± 1.36 | 4.8 ± 0.41 | 6.34 ± 1.02 | 5.83 ± 0.46 |
| Colon | 1.62 ± 0.07 | 1.28 ± 0.09 | 1.15 ± 0.12 | 1.13 ± 0.07 | 1.2 ± 0.11 |
| Colon content | 1.03 ± 0.33 | 1.24 ± 0.14 | 2.55 ± 0.66 | 1.27 ± 0.13 | 2.5 ± 0.76 |
| Ileum | 1.79 ± 0.28 | 4.29 ± 0.65 | 4.67 ± 1.54 | 3.33 ± 0.66 | 4.18 ± 0.63 |
| Ileum content | 0.71 ± 0.33 | 1.37 ± 0.44 | 2.4 ± 0.9 | 1.21 ± 0.26 | 1.59 ± 0.35 |
| Stomach | 2.08 ± 0.16 | 1.49 ± 0.17 | 1.08 ± 0.17 | 1.31 ± 0.05 | 1.35 ± 0.08 |
| Stomach content | 0.34 ± 0.21 | 0.39 ± 0.15 | 0.65 ± 0.21 | 0.48 ± 0.06 | 0.44 ± 0.19 |
| Tail | 1.84 ± 0.30 | 2.44 ± 1.80 | 1.12 ± 0.23 | 1.16 ± 0.08 | 1.91 ± 1.29 |
| Spine | 1.39 ± 0.14 | 2.34 ± 0.28 | 2.39 ± 0.38 | 2.53 ± 0.18 | 3.04 ± 0.43 |
| Brain hemisphere | 0.26 ± 0.05 | 2.54 ± 0.21 | 1.24 ± 0.12 | 1.05 ± 0.06 | 1.13 ± 0.1 |

**Table 5:** Tissue-to-blood ratio of [^89^Zr]Zr-DFO*-mAb conjugates in 10 months old APP/PS1 transgenic mice (TG) or 10 months old WT littermates at d3 after administration of 1 mg/kg of conjugate. Results are expressed as mean ratio to blood ± sd (n=5 mice per group).

| **Organ** | **Adu/TG** | **Adu-8D3/TG** | **Adu-8D3/WT** | **B12-8D3/TG** | **B12-8D3/WT** |
| --- | --- | --- | --- | --- | --- |
| Blood | 1 | 1 | 1 | 1 | 1 |
| Urine | 0.29 ± 0.04 | 1.15 ± 0.25 | 1.49 ± 1.22 | 0.97 ± 0.11 | 1.17 ± 0.54 |
| Skin | 0.24 ± 0.06 | 0.38 ± 0.02 | 0.41± 0.08 | 0.27 ± 0.08 | 0.25 ± 0.03 |
| Bladder | 0.67 ± 0.04 | 1.04 ± 0.13 | 1.20± 0.15 | 0.72 ± 0.04 | 0.69 ± 0.06 |
| Sternum | 0.21 ± 0.01 | 1.28 ± 0.13 | 1.78± 0.40 | 0.90 ± 0.22 | 1.14 ± 0.18 |
| Heart | 0.37 ± 0.02 | 0.51 ± 0.02 | 0.53 ± 0.05 | 0.38 ± 0.03 | 0.37 ± 0.03 |
| Lung | 0.65 ± 0.04 | 0.81 ± 0.07 | 0.93 ± 0.08 | 0.55 ± 0.08 | 0.61 ± 0.07 |
| Liver | 1.81 ± 0.23 | 6.74 ± 1.05 | 9.00 ± 1.45 | 2.45 ± 0.37 | 2.36 ± 0.31 |
| Pancreas | 0.20 ± 0.03 | 0.28 ± 0.07 | 1.97 ± 3.61 | 0.19 ± 0.04 | 0.14 ± 0.02 |
| Spleen | 0.71 ± 0.10 | 24.71 ± 4.84 | 40.70 ± 16.99 | 7.67 ± 0.97 | 7.00 ± 0.73 |
| Kidney L | 1.06 ± 0.12 | 3.75 ± 0.20 | 7.02 ± 3.73 | 1.27 ± 0.08 | 1.25 ± 0.18 |
| Muscle | 0.10 ± 0.02 | 0.12 ± 0.01 | 0.20 ± 0.02 | 0.12 ± 0.02 | 0.13 ± 0.01 |
| Thigh-bone | 0.20 ± 0.05 | 3.49 ± 0.52 | 3.66 ± 0.49 | 2.35 ± 0.46 | 2.15 ± 0.32 |
| Colon | 0.24 ± 0.04 | 0.66 ± 0.03 | 0.87 ± 0.07 | 0.42 ± 0.04 | 0.44 ± 0.03 |
| Colon content | 0.15 ± 0.05 | 0.64 ± 0.08 | 1.95 ± 0.60 | 0.47 ± 0.04 | 0.91 ± 0.27 |
| Ileum | 0.26 ± 0.04 | 2.22 ± 0.33 | 3.61 ± 1.51 | 1.23 ± 0.24 | 1.53 ± 0.19 |
| Ileum content | 0.11 ± 0.05 | 0.72 ± 0.26 | 1.86 ± 0.85 | 0.45 ± 0.11 | 0.59 ± 0.14 |
| Stomach | 0.31 ± 0.02 | 0.77 ± 0.09 | 0.83 ± 0.17 | 0.48 ± 0.02 | 0.50 ± 0.05 |
| Stomach content | 0.05 ± 0.04 | 0.20 ± 0.07 | 0.49 ± 0.16 | 0.18 ± 0.02 | 0.16 ± 0.06 |
| Tail | 0.27 ± 0.05 | 1.23 ± 0.86 | 0.85 ± 0.14 | 0.43 ± 0.05 | 0.73 ± 0.56 |
| Spine | 0.21 ± 0.01 | 1.21 ± 0.09 | 1.82 ± 0.36 | 0.94 ± 0.10 | 1.11 ± 0.16 |
| Brain hemisphere | 0.04 ± 0.01 | 1.32 ± 0.15 | 0.94 ± 0.09 | 0.39 ± 0.02 | 0.41 ± 0.03 |

**Table 6:** Biodistribution of [^89^Zr]Zr-DFO*-mAb conjugates in 10 months old APP/PS1 transgenic mice (TG) or 10 months old WT littermates at d7 after administration of 1 mg/kg of conjugate. Results are expressed as mean (%ID/g) ± sd (n=5 mice per group).

| **Organ** | **Adu/TG** | **Adu-8D3/TG** | **Adu-8D3/WT** | **B12-8D3/TG** | **B12-8D3/WT** |
| --- | --- | --- | --- | --- | --- |
| Blood | 4.49 ± 0.42 | 0.60 ± 0.17 | 0.55 ± 0.09 | 0.88 ± 0.34 | 1.03 ± 0.44 |
| Urine | 1.07 ± 0.22 | 0.64 ± 0.03 | 0.8 ± 0.22 | 0.73 ± 0.38 | 0.64 ± 0.2 |
| Skin | 1.26 ± 0.33 | 0.35 ± 0.12 | 0.27 ± 0.07 | 0.32 ± 0.09 | 0.34 ± 0.12 |
| Bladder | 2.73 ± 0.34 | 1.08 ± 0.20 | 1.02 ± 0.18 | 1.12 ± 0.29 | 0.99 ± 0.14 |
| Sternum | 1.17 ± 0.17 | 1.78 ± 0.46 | 1.99 ± 0.31 | 2.58 ± 1.41 | 2.31 ± 0.54 |
| Heart | 1.59 ± 0.24 | 0.45 ± 0.06 | 0.38 ± 0.02 | 0.6 ± 0.23 | 0.45 ± 0.07 |
| Lung | 3.17 ± 0.37 | 0.63 ± 0.08 | 0.57 ± 0.1 | 0.56 ± 0.14 | 0.74 ± 0.16 |
| Liver | 11.53 ± 3.07 | 10.39 ± 1 | 11.54 ± 0.81 | 7.28 ± 1.29 | 6.91 ± 1.01 |
| Pancreas | 0.79 ± 0.18 | 0.23 ± 0.02 | 0.21 ± 0.07 | 0.24 ± 0.05 | 0.26 ± 0.11 |
| Spleen | 4.55 ± 0.73 | 38.97 ± 4.45 | 49.4 ± 11.24 | 17.25 ± 5.03 | 23.54 ± 7.47 |
| Kidney L | 6.01 ± 0.23 | 5.33 ± 0.88 | 4.73 ± 0.22 | 2.73 ± 0.19 | 2.64 ± 0.52 |
| Muscle | 0.46 ± 0.08 | 0.10 ± 0.01 | 0.1 ± 0.02 | 0.09 ± 0.02 | 0.11 ± 0.02 |
| Thigh-bone | 1.19 ± 0.16 | 4.69 ± 1.58 | 4.96 ± 0.53 | 4.38 ± 0.53 | 4.68 ± 0.98 |
| Colon | 0.89 ± 0.18 | 0.61 ± 0.08 | 0.7 ± 0.09 | 0.52 ± 0.15 | 0.44 ± 0.07 |
| Colon content | 0.90 ± 0.44 | 0.44 ± 0.08 | 0.9 ± 0.33 | 0.57 ± 0.08 | 0.62 ± 0.23 |
| Ileum | 1.12 ± 0.19 | 1.04 ± 0.14 | 1.14 ± 0.27 | 0.78 ± 0.12 | 0.74 ± 0.11 |
| Ileum content | 0.53 ± 0.07 | 0.7 ± 0.15 | 0.73 ± 0.07 | 0.50 ± 0.04 | 0.49 ± 0.12 |
| Stomach | 1.25 ± 0.15 | 0.41 ± 0.05 | 0.41 ± 0.04 | 0.38 ± 0.05 | 0.39 ± 0.07 |
| Stomach content | 0.52 ± 0.72 | 0.11 ± 0.02 | 0.24 ± 0.08 | 0.20 ± 0.07 | 0.17 ± 0.03 |
| Tail | 1.59 ± 0.21 | 1.03 ± 0.15 | 0.92 ± 0.23 | 1.03 ± 0.20 | 1.07 ± 0.50 |
| Spine | 1.10 ± 0.12 | 1.97 ± 0.56 | 2.01 ± 0.1 | 2.24 ± 0.31 | 2.26 ± 0.29 |
| Brain hemisphere | 0.27 ± 0.02 | 1.85 ± 0.17 | 0.83 ± 0.05 | 0.69 ± 0.05 | 0.68 ± 0.17 |

**Table 7:** Tissue-to-blood ratio of [^89^Zr]Zr-DFO*-mAb conjugates in 10 months old APP/PS1 transgenic mice (TG) or 10 months old WT littermates at d7 after administration of 1 mg/kg of conjugate. Results are expressed as mean ratio to blood ± sd (n=5 mice per group).

| **Organ** | **Adu/TG** | **Adu-8D3/TG** | **Adu-8D3/WT** | **B12-8D3/TG** | **B12-8D3/WT** |
| --- | --- | --- | --- | --- | --- |
| Blood | 1 | 1 | 1 | 1 | 1 |
| Urine | 0.24 ± 0.06 | 1.18 ± 0.47 | 1.53 ± 0.61 | 1.02 ± 0.69 | 0.75 ± 0.42 |
| Skin | 0.28 ± 0.07 | 0.60 ± 0.20 | 0.51 ± 0.12 | 0.44 ± 0.28 | 0.35 ± 0.13 |
| Bladder | 0.61 ± 0.02 | 1.88 ± 0.35 | 1.89 ± 0.30 | 1.57 ± 1.08 | 1.05 ± 0.32 |
| Sternum | 0.26 ± 0.02 | 3.05 ± 0.50 | 3.71 ± 0.62 | 3.19 ± 1.49 | 2.47 ± 0.96 |
| Heart | 0.35 ± 0.03 | 0.78 ± 0.17 | 0.70 ± 0.09 | 0.75 ± 0.30 | 0.47 ± 0.10 |
| Lung | 0.70 ± 0.03 | 1.17 ± 0.59 | 1.07 ± 0.27 | 0.79 ± 0.53 | 0.76 ± 0.14 |
| Liver | 2.62 ± 0.90 | 18.47 ± 5.04 | 21.87 ± 5.59 | 11.51 ± 10.85 | 7.54 ± 2.97 |
| Pancreas | 0.18 ± 0.03 | 0.43 ± 0.19 | 0.39 ± 0.11 | 0.32 ± 0.16 | 0.26 ± 0.05 |
| Spleen | 1.02 ± 0.16 | 69.85 ± 21.64 | 91.45 ± 18.49 | 26.91 ± 24.37 | 23.72 ± 3.76 |
| Kidney L | 1.34 ± 0.12 | 9.55 ± 3.26 | 8.91 ± 1.88 | 7.24 ± 7.24 | 2.81 ± 0.97 |
| Muscle | 0.10 ± 0.02 | 0.17 ± 0.04 | 0.18 ± 0.02 | 0.13 ± 0.08 | 0.11 ± 0.03 |
| Thigh-bone | 0.27 ± 0.02 | 7.84 ± 1.07 | 9.39 ± 2.42 | 10.93 ± 10.01 | 5.30 ± 2.61 |
| Colon | 0.20 ± 0.03 | 1.07 ± 0.25 | 1.30 ± 0.19 | 0.72 ± 0.47 | 0.49 ± 0.21 |
| Colon content | 0.20 ± 0.09 | 0.83 ± 0.40 | 1.76 ± 0.92 | 0.85 ± 0.69 | 0.66 ± 0.31 |
| Ileum | 0.25 ± 0.03 | 1.85 ± 0.54 | 2.12 ± 0.57 | 1.09 ± 0.69 | 0.79 ± 0.25 |
| Ileum content | 0.12 ± 0.01 | 1.28 ± 0.56 | 1.38 ± 0.31 | 0.73 ± 0.56 | 0.52 ± 0.18 |
| Stomach | 0.28 ± 0.02 | 0.72 ± 0.18 | 0.76 ± 0.14 | 0.50 ± 0.26 | 0.41 ± 0.11 |
| Stomach content | 0.12 ± 0.15 | 0.19 ± 0.06 | 0.47 ± 0.22 | 0.28 ± 0.21 | 0.18 ± 0.05 |
| Tail | 0.35 ± 0.03 | 1.90 ± 0.83 | 1.72 ± 0.53 | 1.36 ± 0.64 | 1.10 ± 0.56 |
| Spine | 0.25 ± 0.02 | 3.30 ± 0.03 | 3.79 ± 0.83 | 3.15 ± 2.06 | 2.47 ± 0.96 |
| Brain hemisphere | 0.06 ± 0.00 | 3.32 ± 1.08 | 1.57 ± 0.34 | 0.99 ± 0.70 | 0.71 ± 0.22 |

**Dose escalation experiment**

I

III

II

I

III

II

I

**Table 8:** PET images quantification of [^89^Zr]Zr-DFO*-mAb conjugates brain uptake in 10 months old APP/PS1 transgenic mice (TG) at d7 after administration of 30 µg (1 mg/kg), 100 µg, 200 µg and 400 µg of conjugate. Results are expressed as mean (%ID/g) ± sd (n=4 mice per group).

| **Organ** | **Adu-8D3/TG 30** **µg** | **Adu-8D3/TG 100** **µg** | **Adu-8D3/TG 200** **µg** | **Adu-8D3/TG 400** **µg** | **B12-8D3/TG 400** **µg** |
| --- | --- | --- | --- | --- | --- |
| Brain hemisphere | 1.12 ± 0.1 | 0.9 ± 0.18 | 0.76 ± 0.14 | 0.56 ± 0.07 | 0.54 ± 0.05 |

**Table 9:** Biodistribution of [^89^Zr]Zr-DFO*-mAb conjugates in 10 months old APP/PS1 transgenic mice (TG) at d7 after administration of 30 µg (1 mg/kg), 100 µg, 200 µg and 400 µg of conjugate. Results are expressed as mean (%ID/g) ± sd (n=4 mice per group).

| **Organ** | **Adu-8D3/TG 30** **µg** | **Adu-8D3/TG 100** **µg** | **Adu-8D3/TG 200** **µg** | **Adu-8D3/TG 400** **µg** | **B12-8D3/TG 400** **µg** |
| --- | --- | --- | --- | --- | --- |
| Blood | 0.46 ± 0.05 | 0.24 ± 0.09 | 0.25 ± 0.1 | 0.05 ± 0.01 | 0.08 ± 0.02 |
| Urine | 0.58 ± 0.21 | 0.69 ± 0.29 | 0.78 ± 0.23 | 0.68 ± 0.35 | 0.82 ± 0.13 |
| Skin | 0.23 ± 0.05 | 0.25 ± 0.07 | 0.24 ± 0.17 | 0.21 ± 0.07 | 0.21 ± 0.08 |
| Bladder | 0.79 ± 0.12 | 0.71 ± 0.14 | 0.73 ± 0.14 | 0.55 ± 0.08 | 0.79 ± 0.09 |
| Sternum | 1.22 ± 0.30 | 1.59 ± 0.35 | 1.64 ± 0.7 | 1.51 ± 0.26 | 1.17 ± 0.31 |
| Heart | 0.38 ± 0.05 | 0.33 ± 0.04 | 0.4 ± 0.06 | 0.3 ± 0.06 | 0.34 ± 0.04 |
| Lung | 0.57 ± 0.05 | 0.41 ± 0.05 | 0.4 ± 0.1 | 0.33 ± 0.08 | 0.43 ± 0.11 |
| Liver | 19.69 ± 4.76 | 23.68 ± 2.78 | 18.11 ± 3.65 | 21.33 ± 5.33 | 10.04 ± 2.27 |
| Pancreas | 0.17 ± 0.08 | 0.1 ± 0.01 | 0.12 ± 0.01 | 0.1 ± 0.03 | 0.13 ± 0.02 |
| Spleen | 94.4 ± 5.9 | 153.85 ± 82.49 | 50.8 ± 8.8 | 28.14 ± 4.97 | 25.77 ± 5.68 |
| Kidney L | 3.9 ± 0.16 | 3.39 ± 0.35 | 3.28 ± 0.27 | 3.26 ± 0.28 | 3.05 ± 0.33 |
| Muscle | 0.08 ± 0.01 | 0.06 ± 0.01 | 0.08 ± 0.02 | 0.06 ± 0.01 | 0.07 ± 0.02 |
| Thigh-bone | 3.62 ± 0.54 | 4.86 ± 0.65 | 3.45 ± 0.8 | 3.28 ± 0.56 | 3.74 ± 0.71 |
| Colon | 0.5 ± 0.05 | 0.54 ± 0.22 | 0.72 ± 0.11 | 0.55 ± 0.11 | 0.79 ± 0.29 |
| Colon content | 0.57 ± 0.14 | 0.61 ± 0.29 | 0.62 ± 0.28 | 0.29 ± 0.11 | 0.76 ± 0.09 |
| Ileum | 0.98 ± 0.11 | 0.8 ± 0.2 | 0.86 ± 0.14 | 0.69 ± 0.16 | 1 ± 0.13 |
| Ileum content | 0.62 ± 0.19 | 0.51 ± 0.17 | 0.47 ± 0.12 | 0.37 ± 0.07 | 0.62 ± 0.07 |
| Stomach | 0.3 ± 0.03 | 0.47 ± 0.39 | 0.26 ± 0.05 | 0.24 ± 0.04 | 0.31 ± 0.05 |
| Stomach content | 0.11 ± 0.05 | 0.14 ± 0.08 | 0.16 ± 0.13 | 0.06 ± 0.04 | 0.14 ± 0.06 |
| Tail | 0.51 ± 0.08 | 0.58 ± 0.36 | 0.54 ± 0.11 | 0.53 ± 0.26 | 0.53 ± 0.12 |
| Spine | 1.55 ± 0.22 | 1.82 ± 0.47 | 1.49 ± 0.36 | 1.29 ± 0.21 | 1.57 ± 0.43 |
| Brain hemisphere | 1.67 ± 0.18 | 1.33 ± 0.18 | 1 ± 0.14 | 0.71 ± 0.12 | 0.6 ± 0.08 |

**Merged channel Injected Adu-8D3 Thioflavin S**

Adu-8D3/TG

30 μg


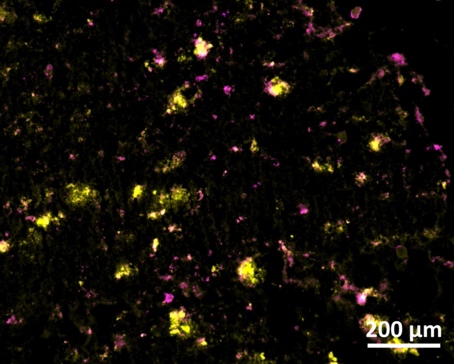

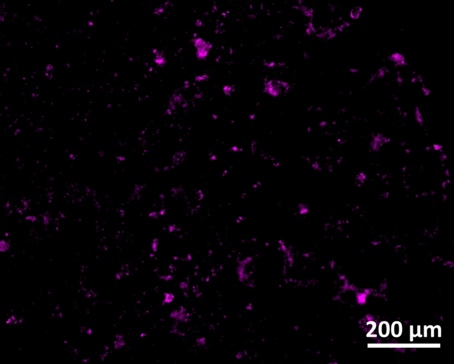

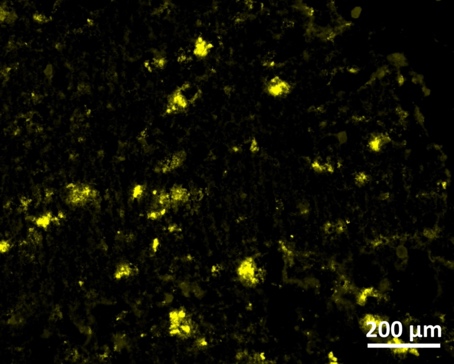


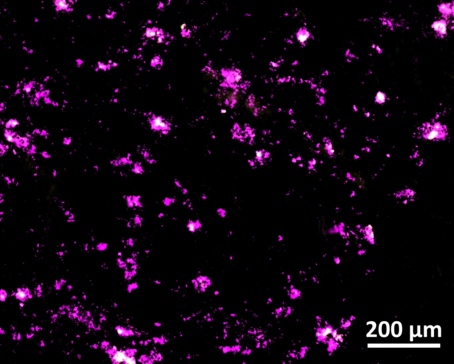

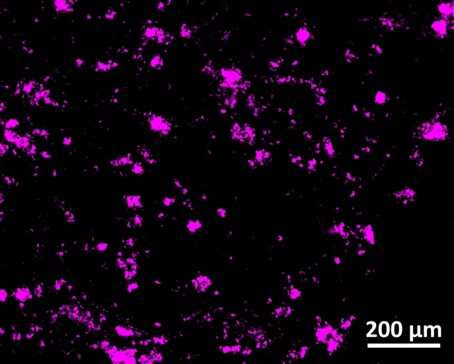

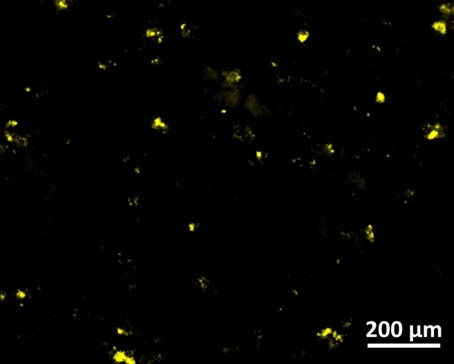


Adu-8D3/TG

100 μg


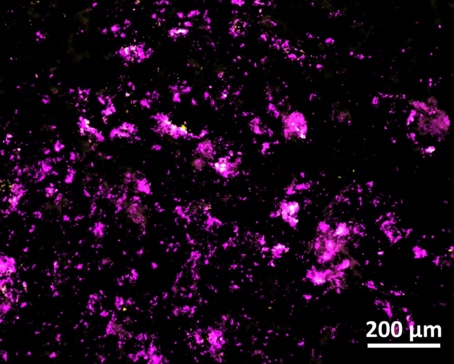

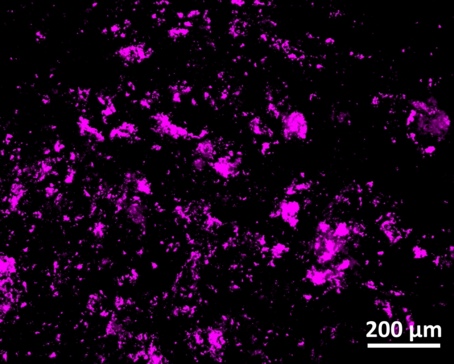

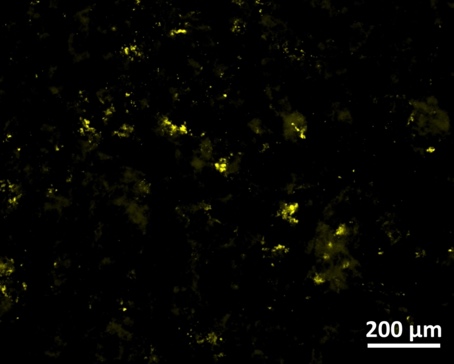


Adu-8D3/TG

200 μg


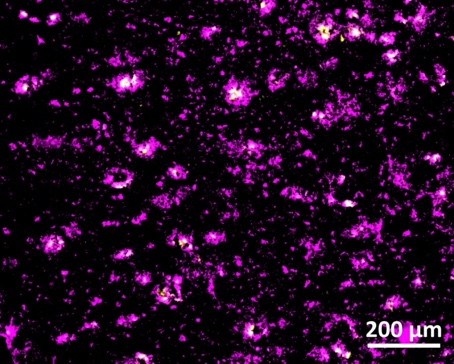

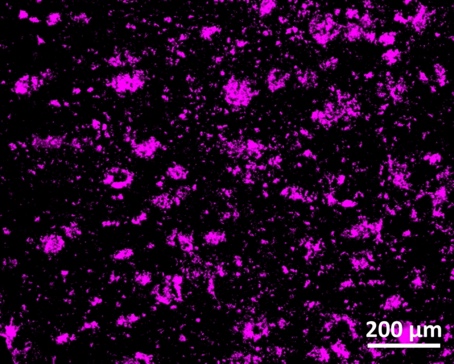

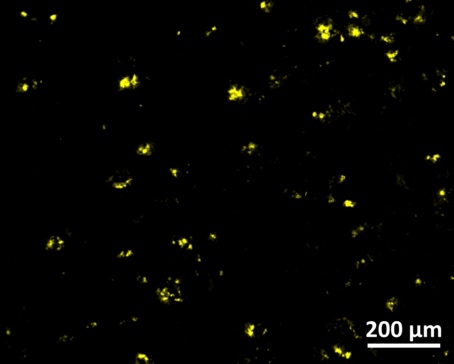


Adu-8D3/TG

400 μg


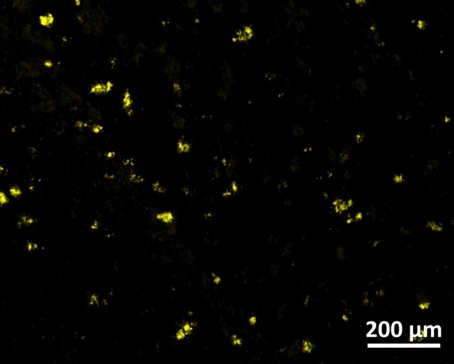

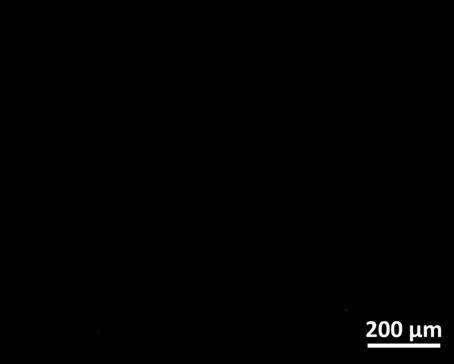

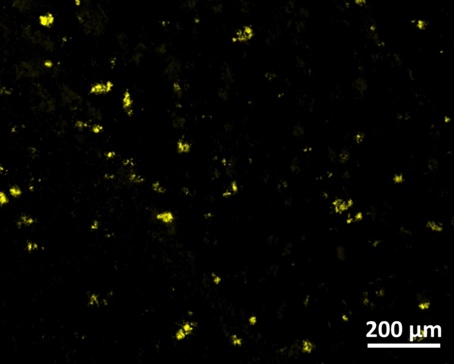


B12-8D3/TG

400 μg

**Figure 3:** *Ex vivo* immunofluorescence staining of Aβ plaques and injected [^89^Zr]Zr-DFO*-Adu-8D3 conjugates (30 μg (1 mg/kg), 100 μg, 200 μg, 400 μg Adu-8D3) and 400 μg B12-8D3) in APP/PS1 transgenic mice (TG) 7 days p.i.. Autoradiography tissue samples were stained with goat anti-human AF647 (1:1000) and Thioflavin S (0.125%). Left panels: merged channels, middle panels: AF647 channel, right panels: AF405 channel (Thioflavin S).

*
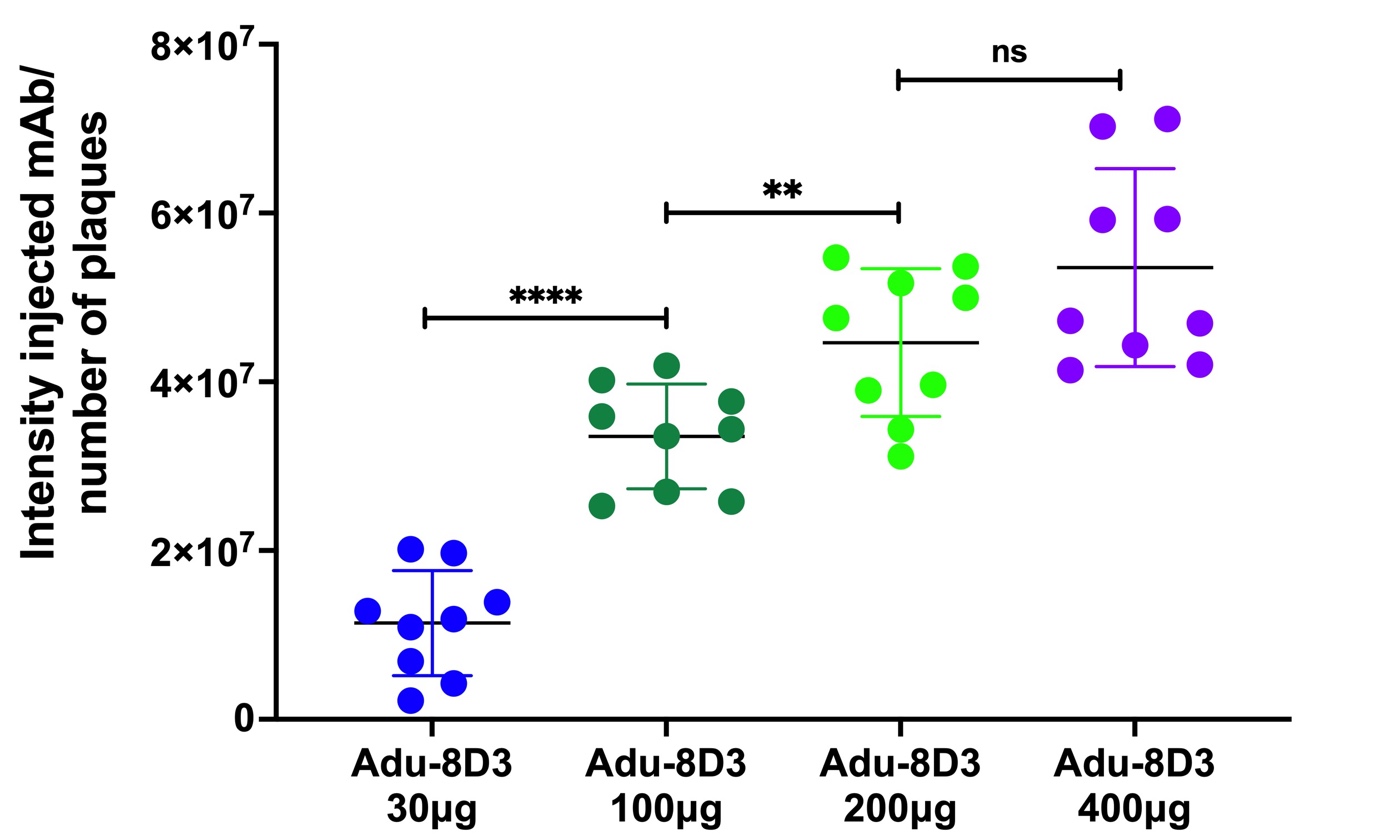
*

**Figure 4:** Quantification of Adu-8D3 in mouse brain at different doses. Thioflavin-S (0.125%) and goat α-human AF647 (1:1000) stained whole brain images were quantified with Zeiss Zen Blue software 3.4 using the cell counting tool of the bio app with the following settings: background subtraction applied, automatic threshold, area 1-3000 μm, circularity 0.0-1.0. 3 mice per group and 3 samples per mice (n=9 per group) were analyzed using 20 μm sagittal cuts of the same brain level (hippocampus visible). The intensity sum of channel AF647 (stained injected antibody) was normalized to the number of plaques (stained with Thioflavin-S). Significant differences between the groups are marked with asterisks (*****p* < 0.0001, ***p* < 0.01); ns: non-significant, *p* = 0.42, analyzed by t-test with Welch’s correction.

**Biodistribution in mice at different age**

**Table 10:** PET images quantification of [^11^C]PIB brain uptake derived from the 5-25 min static frame in 3, 7, 10 months old APP/PS1 transgenic (TG) mice or 3, 7, 10 months old WT littermates. [^11^C]PIB (5 - 10 MBq, molar activity: 72 - 144 GBq/mmol). Results are expressed as mean (%ID/g) ± sd (n=6 mice per group).

| **Organ** | **[^11^C]PIB/TG 3 months** | **[^11^C]PIB/WT 3 months** | **[^11^C]PIB /TG 7 months** | **[^11^C]PIB/WT 7 months** | **[^11^C]PIB/TG 10 months** | **[^11^C]PIB/WT 10 months** |
| --- | --- | --- | --- | --- | --- | --- |
| Brain hemisphere | 2.26 ± 0.57 | 2.09 ± 0.29 | 2.03 ± 0.43 | 1.85 ± 0.59 | 2.7 ± 0.78 | 1.93 ± 0.18 |

**Table 11:** PET images quantification of [^89^Zr]Zr-DFO*-Adu-8D3 brain uptake in 3, 7, 10 months old APP/PS1 transgenic mice (TG) or 3, 7, 10 months old WT littermates, d7 after administration of 1 mg/kg of conjugate. Results are expressed as mean (%ID/g) ± sd (n=6 mice per group).

| **Organ** | **Adu-8D3/TG 3 months** | **Adu-8D3/WT 3 months** | **Adu-8D3/TG 7 months** | **Adu-8D3/WT 7 months** | **Adu-8D3/TG 10 months** | **Adu-8D3/WT 10 months** |
| --- | --- | --- | --- | --- | --- | --- |
| Brain hemisphere | 1.34 ± 0.24 | 1.14 ± 0.15 | 1.41 ± 0.26 | 0.87 ± 0.17 | 1.32 ± 0.15 | 0.8 ± 0.1 |

**Table 12:** Biodistribution of [^89^Zr]Zr-DFO*-Adu-8D3 in 3, 7, 10 months old APP/PS1 transgenic mice (TG) or 3, 7, 10 months old WT littermates at d7 after administration of 1 mg/kg of conjugate. Results are expressed as mean (%ID/g) ± sd (n=6 mice per group).

| **Organ** | **Adu-8D3/TG 3 months** | **Adu-8D3/WT 3 months** | **Adu-8D3/TG 7 months** | **Adu-8D3/WT 7 months** | **Adu-8D3/TG 10 months** | **Adu-8D3/WT 10 months** |
| --- | --- | --- | --- | --- | --- | --- |
| Blood | 0.84 ± 0.08 | 0.86 ± 0.09 | 0.59 ± 0.09 | 0.7 ± 0.11 | 0.57 ± 0.07 | 0.49 ± 0.24 |
| Urine | 0.72 ± 0.15 | 0.68 ± 0.41 | 0.62 ± 0.23 | 0.72 ± 0.22 | 0.67 ± 0.16 | 1.13 ± 0.57 |
| Skin | 0.51 ± 0.2 | 0.71 ± 0.12 | 0.45 ± 0.14 | 0.47 ± 0.09 | 0.47 ± 0.03 | 0.49 ± 0.14 |
| Bladder | 1.06 ± 0.19 | 1.32 ± 0.14 | 0.94 ± 0.14 | 1.01 ± 0.13 | 1.07 ± 0.22 | 1.1 ± 0.14 |
| Sternum | 1.61 ± 0.24 | 1.64 ± 0.26 | 1.72 ± 0.12 | 2.07 ± 0.42 | 1.92 ± 0.27 | 2.04 ± 0.4 |
| Heart | 0.74 ± 0.13 | 0.81 ± 0.07 | 0.58 ± 0.08 | 0.67 ± 0.08 | 0.49 ± 0.11 | 0.57 ± 0.11 |
| Lung | 0.52 ± 0.07 | 0.53 ± 0.06 | 0.44 ± 0.04 | 0.45 ± 0.04 | 0.45 ± 0.03 | 0.44 ± 0.08 |
| Liver | 7.59 ± 1.36 | 7.83 ± 1.05 | 10.69 ± 0.92 | 10.43 ± 2.44 | 10.99 ± 1.74 | 13.98 ± 3.75 |
| Pancreas | 0.23 ± 0.06 | 0.21 ± 0.03 | 0.19 ± 0.03 | 0.2 ± 0.03 | 0.2 ± 0.02 | 0.22 ± 0.03 |
| Spleen | 51.95 ± 18.64 | 52.64 ± 11.45 | 48.85 ± 9.13 | 48.77 ± 24.87 | 48.99 ± 15.67 | 45.6 ± 15.9 |
| Kidney L | 5.02 ± 0.18 | 5.14 ± 0.66 | 5.06 ± 0.35 | 5.65 ± 0.65 | 5.8 ± 0.84 | 5.67 ± 0.45 |
| Muscle | 0.13 ± 0.04 | 0.15 ± 0.02 | 0.11 ± 0.01 | 0.1 ± 0.01 | 0.09 ± 0.01 | 0.1 ± 0.02 |
| Thigh-bone | 3.96 ± 0.43 | 4.17 ± 0.77 | 4.34 ± 0.69 | 4.12 ± 0.55 | 3.6 ± 0.34 | 3.68 ± 0.56 |
| Colon | 0.72 ± 0.25 | 0.75 ± 0.33 | 0.53 ± 0.15 | 0.7 ± 0.14 | 0.42 ± 0.07 | 0.6 ± 0.13 |
| Colon content | 0.54 ± 0.18 | 0.83 ± 0.19 | 0.46 ± 0.1 | 0.72 ± 0.12 | 0.4 ± 0.1 | 0.66 ± 0.27 |
| Ileum | 1.03 ± 0.59 | 1.3 ± 0.15 | 1.14 ± 0.2 | 1.29 ± 0.23 | 1.22 ± 0.24 | 1.02 ± 0.3 |
| Ileum content | 0.57 ± 0.17 | 0.6 ± 0.06 | 0.63 ± 0.17 | 0.65 ± 0.09 | 0.55 ± 0.13 | 0.66 ± 0.26 |
| Stomach | 0.5 ± 0.02 | 0.5 ± 0.05 | 0.39 ± 0.07 | 0.46 ± 0.09 | 0.41 ± 0.04 | 0.43 ± 0.08 |
| Stomach content | 0.14 ± 0.05 | 0.14 ± 0.07 | 0.06 ± 0.03 | 0.15 ± 0.05 | 0.11 ± 0.05 | 0.11 ± 0.06 |
| Tail | 1.23 ± 0.59 | 1.71 ± 0.92 | 1.14 ± 0.42 | 1.32 ± 0.93 | 1.46 ± 0.64 | 0.79 ± 0.29 |
| Spine | 1.86 ± 0.2 | 1.86 ± 0.16 | 1.86 ± 0.17 | 2.04 ± 0.32 | 1.91 ± 0.13 | 1.93 ± 0.3 |
| Brain hemisphere | 1.63 ± 0.11 | 1.27 ± 0.13 | 1.89 ± 0.16 | 1 ± 0.17 | 1.57 ± 0.17 | 0.76 ± 0.08 |

**Merged channel Injected Adu-8D3 Thioflavin S**


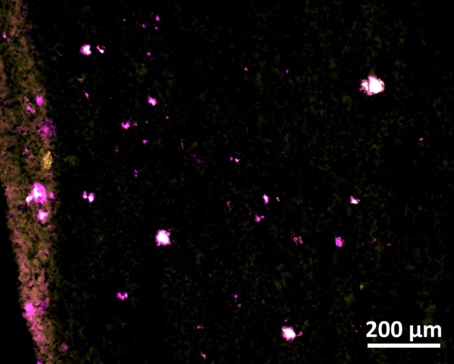

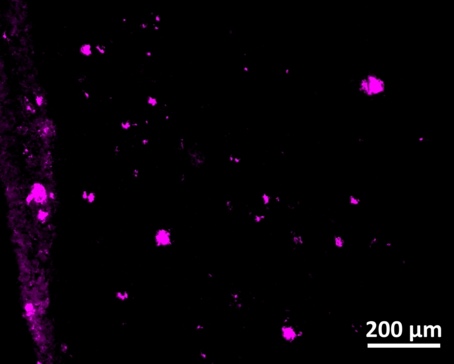

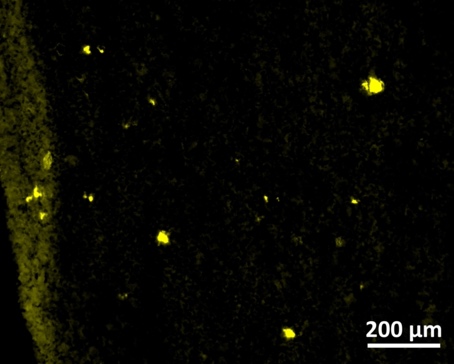


Adu-8D3/TG

3 months


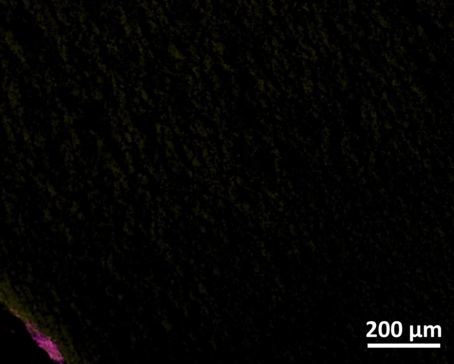

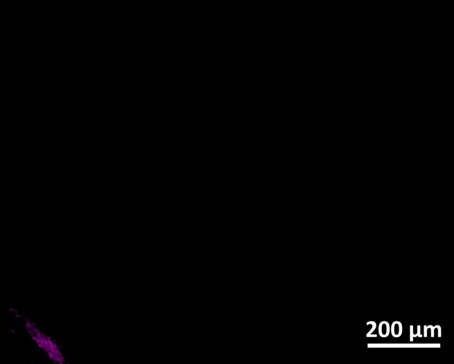

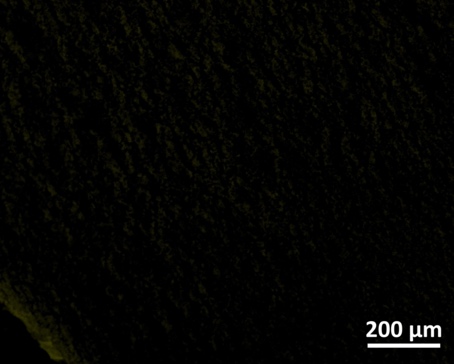


Adu-8D3/WT

3 months


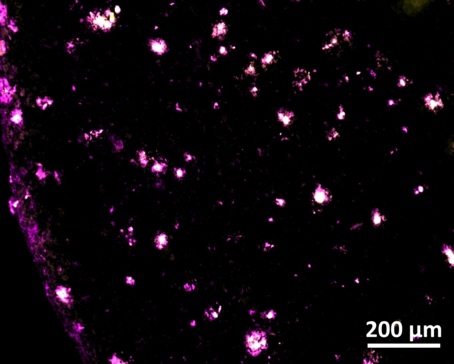

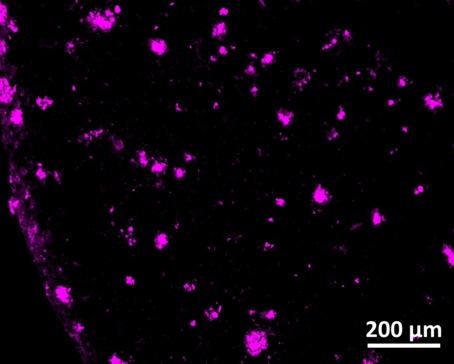

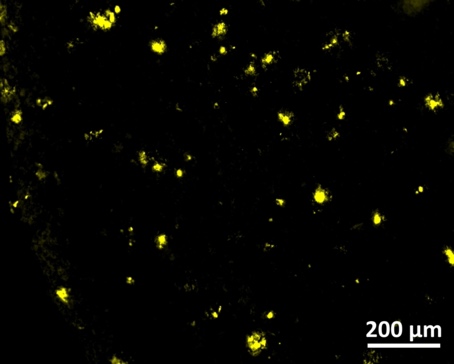


Adu-8D3//TG

7 months


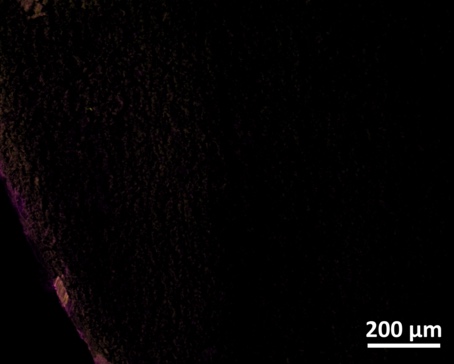

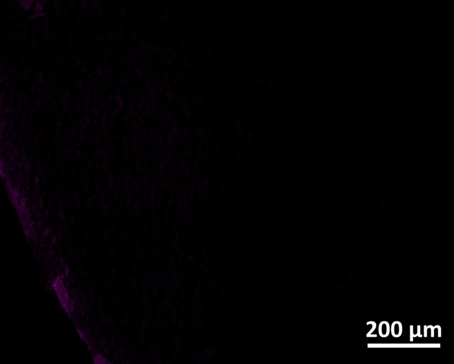

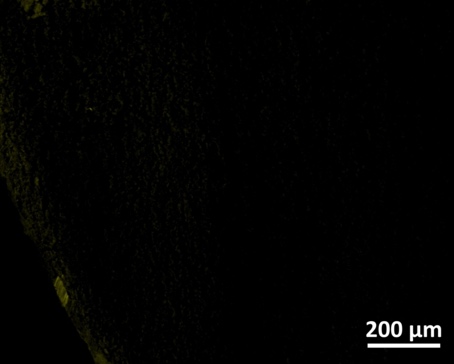


Adu-8D3//WT

7 months


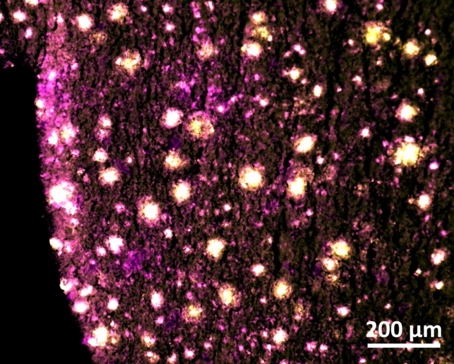

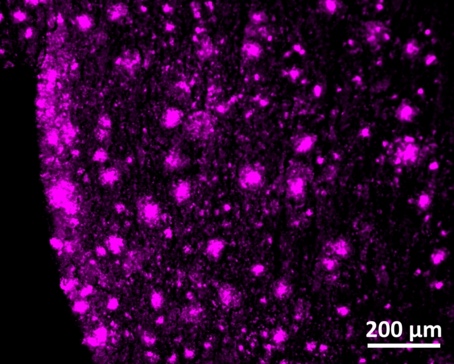

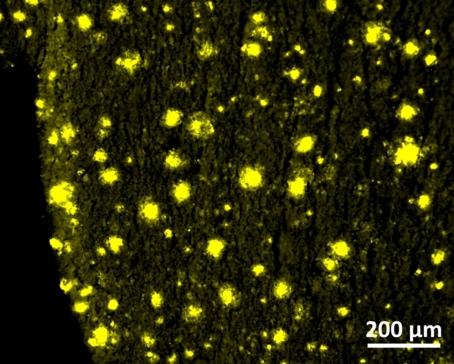


Adu-8D3//TG

10 months


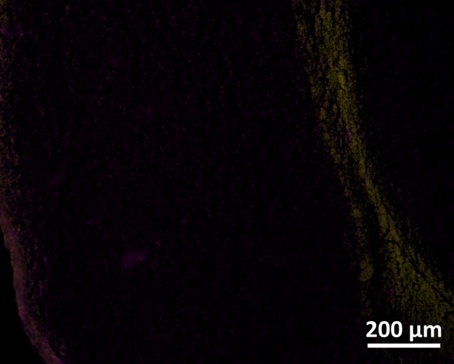

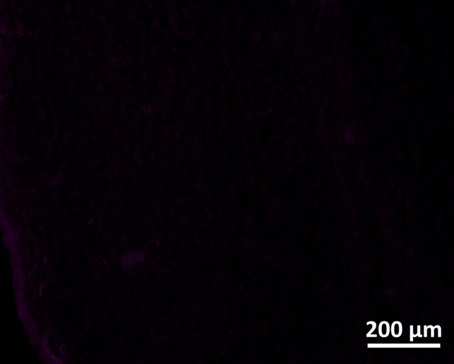

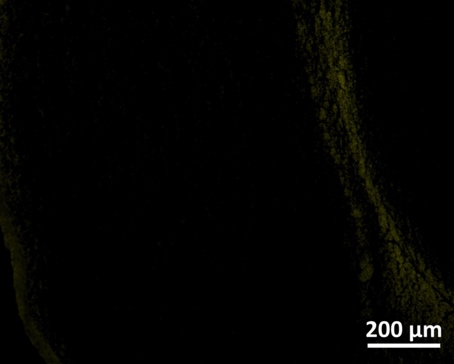


Adu-8D3//WT

10 months

**Figure 5:** *Ex vivo* immunofluorescence staining of Aβ plaques and injected [^89^Zr]Zr-DFO*-Adu-8D3 conjugates (1 mg/kg) in 3 months, 7 months and 10 months old APP/PS1 transgenic mice (TG) and WT littermates 7 days p.i. Autoradiography tissue samples were stained with goat anti-human AF647 (1:1000) and Thioflavin S (0.125%). Left panels: merged channels, middle panels: AF647 channel, right panels: AF405 channel (Thioflavin S).


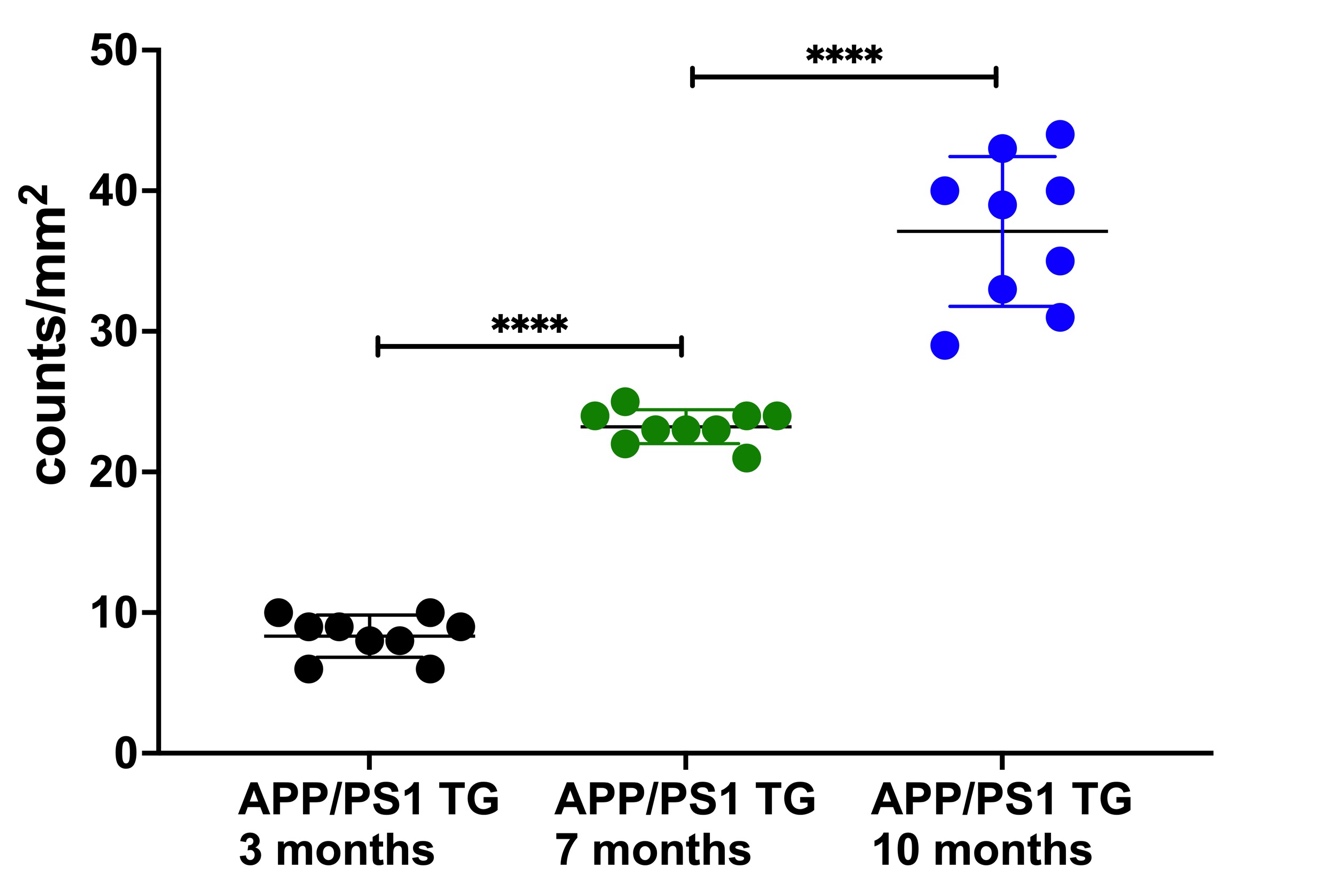


**Figure 6:** Quantification of brain Aβ plaque load for different ages of APP/PS1 transgenic mice (TG). Thioflavin-S (0.125%) stained whole brain images were quantified with Zeiss Zen Blue

software 3.4 using the cell counting tool of the bio app with the following settings: background subtraction applied, automatic threshold, area 1-3000 μm, circularity 0.0-1.0. 3 mice per group and 3 samples per mice (n=9 per group) were analyzed using 20 μm sagittal cuts of the same brain level (hippocampus visible). Significant differences between the groups are marked with asterisks (*****p* < 0.0001) analyzed by t-test with Welch’s correction.


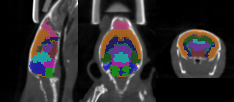


**Figure 7:** Figure showing the region of interest drawn for the PET scans analysis. Vivoquant atlas was applied to delineate the brain.


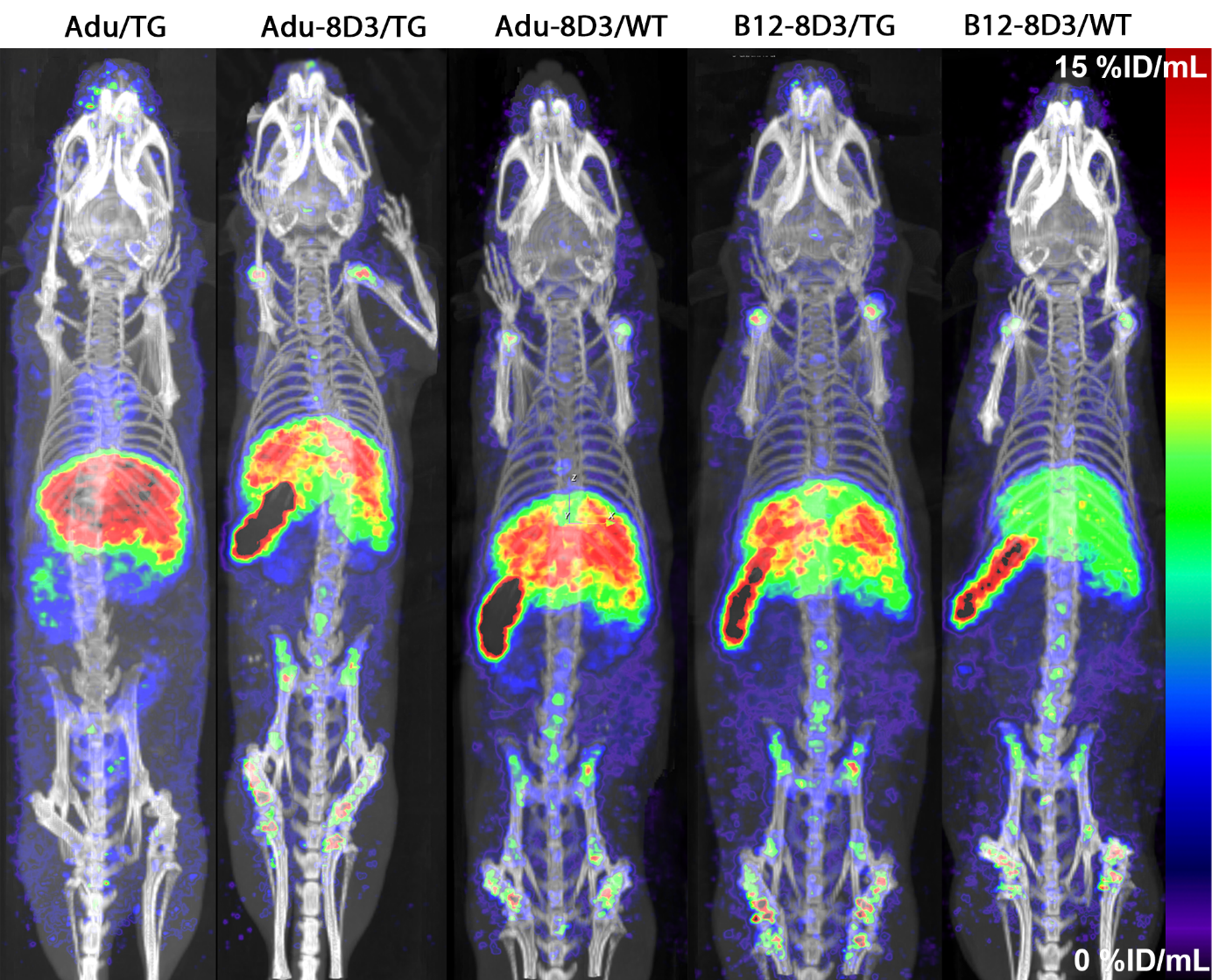


**Figure 8:** Representative maximum image projection (MIP) PET images showing full body uptake of Adu, Adu-8D3 and B12-8D3 in TG and WT mice at d7 p.i.

**[^11^C]PIB PET imaging**

For [^11^C]PIB dynamic PET scans were acquired immediately after i.v. administration of [^11^C]PIB (5 - 10 MBq, molar activity: 72 - 144 GBq/μmol) via a tail vein catheter. A 5 min CT scan or a 12 min multi-FOV MRI scan were acquired after each PET scan and used for attenuation and scatter correction purposes. For [^11^C]PIB, dynamic PET scans were reconstructed into the following frame sequence: 4 × 5, 4 × 10, 2 × 30, 3 × 60, 2 × 300, 1 × 600, 1 × 900, and 1 × 1200 s. Static reconstruction of the 5 – 25 min frame was also performed to obtain static PET images.

**
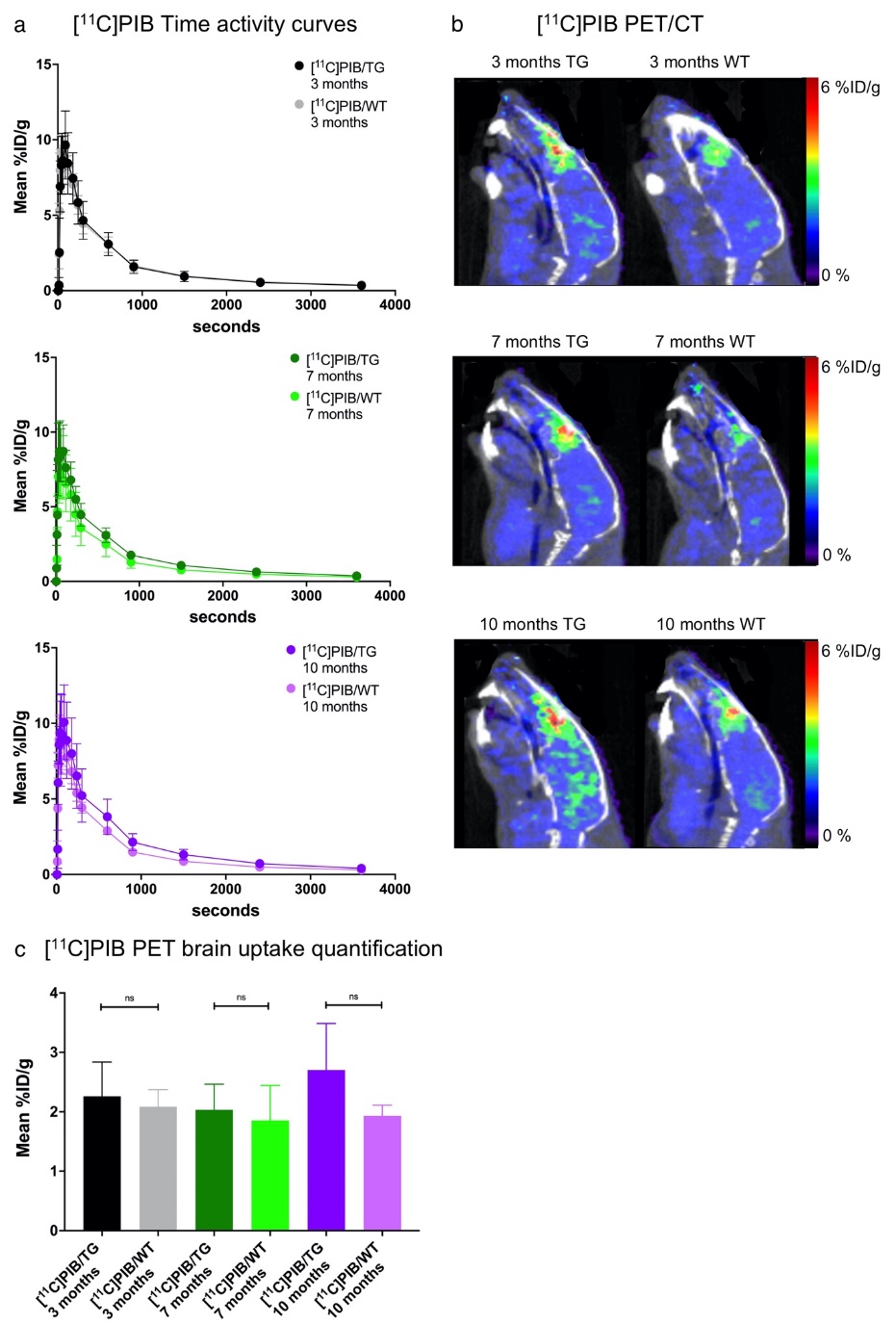
**

**Figure 9:** Aβ imaging of different plaque loads with [^11^C]PIB in APP/PS1 TG and WT littermates at ages 3, 7, and 10 months. a) Time activity curves of brain uptake of [^11^C]PIB; b) Representative sagittal PET images extracted from the static reconstruction of the 5–25 min frame and showing [^11^C]PIB uptake in the brain; c) Quantification of brain uptake of a single frame static reconstruction between 5 and 25 min. Brain uptake is expressed as %ID/g (mean ± SD, n = 3 animals per group). ns: non-significant, p > 0.2, analyzed via t-test with Welch correction.
